# Differentiation of ecological niche patterns between sympatric lemurs in northwestern Madagascar: Implications for their conservation

**DOI:** 10.1371/journal.pone.0345256

**Published:** 2026-03-19

**Authors:** Fernando Mercado Malabet, Finaritra T. Randimbiarison, Jean Claude Razafimampiandra, Bertrand Andriatsitohaina, Coral Chell, Mamy Razafitsalama, Travis S. Steffens, Shawn M. Lehman

**Affiliations:** 1 Department of Anthropology, University of Toronto, Toronto, Ontario, Canada; 2 Mention Zoologie et Biodiversité Animale, Université d’Antananarivo, Antananarivo, Madagascar; 3 Faculté des Sciences, de Technologies et de l’Environnement, Université de Mahajanga, Mahajanga, Madagascar; 4 Department of Sociology and Anthropology, University of Guelph, Guelph, Ontario, Canada; 5 Planet Madagascar, Guelph, Ontario, Canada; Tsinghua University, CHINA

## Abstract

Understanding how species respond to habitat loss and fragmentation is a critical requirement for effective conservation action, particularly in biodiversity hotspots like Madagascar. Species with specialized, narrower ecological niche requirements are hypothesized to be more vulnerable to extinction than generalists, yet empirical tests of this prediction among closely related taxa remain limited. Here, we compare the ecological niche patterns and predicted distributions of two sympatric lemurs in northwestern Madagascar – the Vulnerable Common Brown Lemur (*Eulemur fulvus*) and the Critically Endangered Mongoose Lemur (*Eulemur mongoz*) – to assess how niche flexibility relates to extinction risk. Using presence-only data collected between 2015 and 2020 and ten environmental covariates, we developed species distribution models and ran niche equivalence analysis. The models indicate that *E. fulvus* occupies a broader and more continuous predicted distribution range (48,591 ha) than *E. mongoz* (17,757 ha). In comparison, *E. mongoz* is predicted to occur primarily in moist lowland forests near water basins, showing a stronger spatial association with these habitat conditions that *E. fulvus*. Despite these marked differences in their predicted geographic distributions, niche equivalence analysis showed substantial overlap in the environmental conditions occupied by the two species within the study area. Together, these results suggest that *E. mongoz’s* restricted distribution is not explained solely by the measured environmental predictors, highlighting the need for future work that integrates additional environmental variables and evaluates potential behavioural or demographic constraints not captured here. These findings highlight how subtle differences in niche requirements can shape a species’ habitat use and vulnerability to environmental change. From a management perspective, our findings support prioritizing the protection of moist lowland forests near water basins for *E. mongoz* while maintaining or enhancing habitat connectivity for *E. fulvus* in fragmented landscapes.

## 1. Introduction

Variability in species’ ecological niche requirements is critical determinant of a species’ vulnerability to extinction [1–8]. A niche encompasses the full range of abiotic conditions (e.g., climate, elevation, water availability, habitat availability/quality) that are fundamental to its survival within a specific geographic area, as well as the biotic characteristics (e.g., competition, dietary preferences, locomotion) that restrict their use of the local environment [9–12]. Thus, a species’ niche describes their specific relationship with an ecosystem [9,10,12]. From a biogeographic perspective, these requirements act as environmental filters, excluding species with traits that are incompatible with local conditions [13–17]. This framework underpins the generalist-specialist hypothesis [8,18]: Species with relatively broader niche requirements – ecological generalists – are more likely to tolerate environmental change and persist across a wider range of habitats. In comparison, ecological specialists with narrower niche requirements are more likely to be filtered out under changing or degraded conditions, making them more vulnerable to extinction [8,18–20]. Understanding a species’ niche requirements is therefore important to predicting its ecological flexibility and assessing extinction risk.

Disturbed landscapes – characterized by habitat loss, fragmentation, degradation, and anthropogenic disturbance – intensify the filtering process on species’ persistence [13,17,18,21]. These landscapes often exhibit increased heterogeneity in biotic and abiotic conditions, favouring generalist species due to their capacity to exploit a wide range of environmental conditions and maintain connectivity across fragmented systems [8,18,21–23]. In comparison, specialists are constrained by limited habitat options and reduced patch connectivity [8,10,20,24], facing heightened demographic risks because individuals experience limited opportunities to locate new suitable habitat and mates during dispersal [13,25–28]. These pressures are compounded by additional challenges, such as increased competition from generalists or invasive species that are better suited to the altered conditions [12,29,30]. These dynamics reinforce the link between niche breadth and extinction vulnerability, as specialists experience greater isolation and population decline when suitable habitats shrink or become disconnected [24,31–33].

For arboreal mammals, particularly primates, niche breath can strongly influence their persistence in disturbed and fragmented landscapes [10,14,21]. Generalist species, with broader ecological tolerances, are more likely to encounter a higher amount of suitable habitat patches across the landscape [8,24,34]. In fragmented systems, the surrounding matrix can also serve as a functional extension of their available habitat – providing lower-quality but usable secondary habitat or facilitating movement opportunities between patches [23,24,35–37]. In this context, the effectiveness of matrix elements often depends on their structural similarity to preferred habitats, helping to reduce the costs of dispersal and enhance connectivity [36]. Conversely, specialists, marked by narrow niche requirements, are restricted to fewer habitat types and experience reduced permeability across the matrix, elevating their extinction risk in disturbed habitats [8,18,24,37–39]. A meta-analysis by Galán-Acedo et al. [40] found that primates capable of using non-forest landcover types – such as grasslands or human settlements – for foraging or traveling were significantly more likely to maintain stable or increasing populations. Examples include Vervet Monkeys (*Chlorocebus pygerythrus*) and Rhesus Macaques (*Macaca mulatta*), as both species thrive in human-modified landscapes [41,42], unlike strictly arboreal forest specialists that are far less tolerant of heavily modified environments.

Primates are an ideal taxonomic group for investigating how subtle differences in the niche characteristics of closely related species influence variation in their extinction risk. Previous research has shown that primates tend to exhibit highly similar ecological niche profiles, with greater niche similarity observed within biogeographic regions than between them [12,43]. Understanding the relationship between niche breadth and vulnerability is especially relevant for lemurs in Madagascar – one of the world’s most important biodiversity hotspots. [44,45]. Lemurs represent one of the most threatened primate groups, with all 112 extant species and subspecies experiencing population declines and approximately 95% classified as being near or under the threat of extinction [46–49]. This is particularly concerning given that Madagascar has lost 44% of its forest cover between 1953 and 2014, and much of what remains is highly fragmented [50,51]. While lemurs as a group are highly vulnerable to habitat disturbance, extinction risk varies remarkedly among species – even among sympatric congeners – reflecting not only differences in environmental threat intensity but also intrinsic traits such as niche breadth, body size, generation time, and social structure [3, 4, 46, 52–56]. For example, in northwestern Madagascar, sympatric populations of *Microcebus murinus* (Least Concern) and *Microcebus ravelobensis* (Vulnerable) respond differently to fragmentation: *M. murinus* can use matrix habitats as secondary habitats or effective dispersal mediums, whereas *M. ravelobensis* remains clustered within forest cores, avoiding edge and matrix zones [57]. These differences underscore the importance of niche requirements in shaping species’ resilience to habitat disturbance and, ultimately, their risk of extinction.

**Fig 1:**
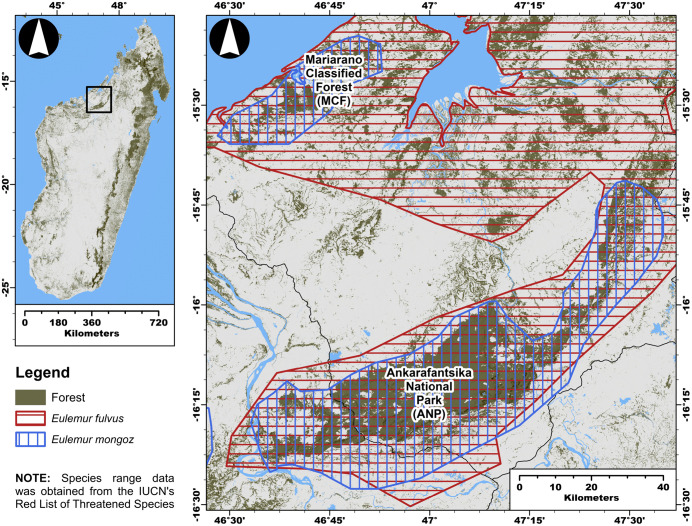
Geographic distribution of sympatric E. fulvus and E. mongoz across dry-deciduous forests in the Boeny region, northwestern Madagascar. Also marked are the locations of Mariarano Classified Forest (MCF) and Ankarafantsika National Park (ANP). Map includes species ranges reprinted from the IUCN Red List of Threatened Species™ under a CC BY 4.0 license, with permission from IUCN, original copyright IUCN, 2024.

Our study focuses on two other sympatric species – the Vulnerable Common Brown Lemur (*Eulemur fulvus*) [58] and the Critically Endangered Mongoose Lemur (*Eulemur mongoz*) [59] – within fragmented dry-deciduous forest landscapes of the Boeny region, west of the Betsiboka River. These landscapes are comprised by seasonal dry-deciduous tropical forests embedded in a mixed-use matrix of grassland and cropland (Fig 1) [60–64]. Both species are arboreal, cathemeral folivore-frugivores of similar body mass (2–4 kg) that coexist within the same forest systems [52,65,66], making them well-suited for comparative analysis of how subtle differences in niche requirements shape responses to habitat disturbance.

Despite their ecological similarities, *Eulemur* species exhibit key differences that influence their niche patterns and affect how they each respond to habitat loss, fragmentation, and deterioration. *E. fulvus* and *E. mongoz* both exhibit long-term stable sympatry in the northwest of Madagascar, which has been associated with seasonal shifts in activity patterns – *E. mongoz* tends to be more nocturnal during the dry season, particularly in disturbed patches, likely due to their lower competitive ability and seasonal variation in fruit availability [66–68]. These species also differ in social structure and space use. Specifically, *E. fulvus* live in large multi-male multi-female groups, composed of 3–15 individuals, and occupy overlapping home ranges (7–20 ha) [58,69]. In comparison, *E. mongoz* forms small, pair-bonded groups that defend smaller, more stable territories (1-5.5 ha), requiring consistent resource availability [70–72]. Additionally, *E. mongoz* is exclusively found in the lowland basins of dry-deciduous forest [59,73] whereas *E. fulvus* is found across a larger range of environmental conditions in dry-deciduous forests in the northwest of Madagascar as well as medium-altitude and lowland moist evergreen rainforests in the east [58,63] – employing a shifting range strategy that tracks the seasonal availability of resources across their home range [66,69,74,75]. These ecological and behavioral differences suggest that variation in the niche requirements of these two species – particularly their habitat tolerance and foraging strategies – may underlie the contrasting extinction risks.

### 1.1. Objectives and hypotheses

Our objective was to determine whether differences in the ecological niches of *E. fulvus* and *E. mongoz* across fragmented landscapes in northwestern Madagascar help explain variation in their extinction risk by comparing the size, connectivity, and environmental breadth of their predicted distributions across geographic and environmental space. We tested two hypotheses:

*H1:* In areas of sympatry, Vulnerable *E. fulvus* will exhibit larger and more continuous areas of predicted occurrence compared to the smaller, more fragmented distributions of Critically Endangered *E. mongoz*.

*H2:* Vulnerable *E. fulvus* will occupy a broader and more generalized niche space relative to the more specialized niche requirements of Critically Endangered *E. mongoz*.

## 2. Materials and methods

To investigate how ecological differences influence species’ vulnerability to habitat disturbance, we modeled and compared the predicted distributions and ecological niche patterns of *E. fulvus* and *E. mongoz* across two dry-deciduous forest landscapes in northwestern Madagascar where they occur in sympatry. Our approach follows previous ecological niche modeling (ENM) studies of other lemur taxa (e.g., *Eulemur*, *Microcebus*, *Cheirogaleus*, *Phaner*), which have provided valuable insights into species’ population ecology, thus informing our methodological framework [76–79]. We developed distribution models (SDMs) [80,81] of *E. fulvus* and *E. mongoz* from occurrence records and 10 independent environmental covariates, to quantify and compare the predicted distributions of *E. fulvus* and *E. mongoz* across two major dry-deciduous forest landscapes where they occur in sympatry. We then assessed the degree of niche overlap and divergence [82] between these two species to evaluate how differences in habitat tolerance reflect ecological mechanisms of environmental filtering or competitive pressure, and correspond to their current conservation status. For this assessment, we assumed that current IUCN Red List assessments accurately reflect population trends within our study region. Both species are considered threatened due to ongoing habitat loss and unsustainable hunting, though the severity of population decline differs between them [58,59].

### 2.1. Landscape context

We surveyed populations of *E. fulvus* and *E. mongoz* in the Mariarano Classified Forest (MCF) and in the western portion of Ankarafantsika National Park (ANP), northwestern Madagascar (Fig 1). MCF and ANP are located approximately 100 km apart, separated by anthropogenically dominated landscapes that include human settlements, agricultural fields, and grasslands. The habitat structure of MCF and ANP can be generally summarized as a fragmented network of small forest patches that surround the periphery of the larger remaining continuous forest. In both landscapes, fragmented forests are embedded in a heterogenous matrix of secondary forests, cropland, and grassland habitats. Both landscapes are part of the dry-deciduous forest ecoregion of Madagascar, which is the most threatened type of forest ecosystem on the island [63]. Dry-deciduous forest landscapes are commonly threatened by high rates of forest loss and fragmentation that are the result of wildfires caused by slash-and-burn agriculture, cyclones, and seasonal drought periods [63,64,83]. Furthermore, these landscapes are two of the largest bodies of continuous forest habitats where *E. fulvus* and *E. mongoz* can be found in sympatry in northwestern Madagascar (Fig 1).

At MCF, the remaining forest habitats are located at low altitudes (~10–100 m asl) and exhibit a high range of vegetation moisture due to the high concentration of surface water bodies and the proximity of this landscape to the Indian Ocean. In contrast, forests at ANP are generally located at higher elevations (~25–300 m asl) and exhibit lower moisture levels than at MCF due to restrictions of the spatial distribution of surface water bodies to the valleys that mark the topography of this landscape. Human influence at MCF and ANP is ubiquitous, driven by the presence of several villages located in the vicinity of the remaining forest, their agricultural activities, and network of roads that interconnect them. Both landscapes are actively managed by their own conservation authorities. MCF is managed by the Vondron’Olona Ifotony (VOI), a community-based forest protection program, while ANP is managed by Madagascar National Park (MNP), in partnership with various conservation NGOs – including Planet Madagascar, Durrell Wildlife Conservation Trust, and Eden: People + Planet.

### 2.2. Sampling of occurrence records

Collection of occurrence records for *E. fulvus* and *E. mongoz* was completed during the dry season (May to October), between 2018 and 2021. We restricted sampling to the dry season because flooding during the rainy season prevents road access to most sites, and because dry-season conditions reflect peak resource limitations (i.e., lowest vegetation greenness/activity), yielding conservative estimates of suitable habitat extent. During the 2018 and 2019 seasons, occurrence records were collected in each landscape by using a repeated visits approach to confirm each species’ occupancy. Repeated-visit surveys were completed by regularly searching the 500 m buffer area surrounding existing trails and newly marked transect tracks. To the best of our abilities, neighbouring transects were marked parallel to each other on an east-to-west direction and covered a length between 500 m to 1 km. Though slight deviations from this pattern occurred because of patch shape or topography, no transect was placed perpendicularly to a neighbouring one within the same landscape. Each transect was separated by at least 500 m from the next closest tract. A total of 20 transects were surveyed between the two landscapes, 9 at MCF and 11 at ANP. At ANP, transects and existing trails extended across forests located in the vicinity of the Ampijoroa research station, and the villages of Ambarindahy, Maevatanimbary, and Andranohobaka (S1 Fig). At MCF, transects and existing trails covered forests near the Mariarano village, as well as the Matsedroy and Antafiameva research stations (S1 Fig). To maximize the chances of encounter for each species, each existing trail and newly marked transect was surveyed a total of three times. All surveys during these two seasons took place either in the afternoon (between 14:00 and 18:00 hours) or evening (between 20:00 and 00:00 hours), when both species were most active. Additional occupancy area surveys were completed at ANP between 2020 and 2021 by patrolling teams from Planet Madagascar (PM), a community-based conservation NGO that works in the park. PM teams were composed of local Malagasy villagers that reside in the park, trained by primatologists from the University of Guelph (T.S. Steffens) and Université de Mahajanga (B. Andriatsitohaina) who have extensive research experience in the region and are adept at spotting and identifying lemurs in the park. These area-based surveyed were completed to maximize sampling coverage in ANP. These surveys tracked populations of *E. fulvus* and *E. mongoz* in forest sites surrounding the villages of Ambarindahy, Maevatanimbary, and Andranohobaka, as well as the Ambanjabe Forest Fragment site (AFFS). Whenever we observed groups or individuals from either *E. fulvus* or *E. mongoz*, we recorded the coordinates of their locations using a handheld GPS, the species ID and site ID, and the time of observation.

All field protocols for the survey of *E. fulvus* and *E. mongoz* were reviewed and approved by the appropriate ethics committees at the University of Toronto, Canada (Protocol # 20012385). Our field protocols adhered to the American Society of Primatologists (ASP) Principles for the Ethical Treatment of Non-Human Primates (https://www.asp.org/society) and the American Society of Primatologists Code of Best Practices for Field Primatology (https://www.asp.org/resources). All fieldwork adhered to the legal requirements of Madagascar and were approved by the Direction de la Gestion des Ressources Naturelles Renouvelables et des Ecosystemes and by Madagascar National Parks (Permits #104/18/MEEF/SG/DGF/DSAP/SCB.Re and #115/19/MEDD/SG/DGEF/DGRNE).

### 2.3. Environmental covariates

We used ten large-scale measures of habitat quality and landscape composition to model the distribution patterns of *E. fulvus* and *E. mongoz* in northwestern Madagascar and compare their niche equivalence patterns. These environmental covariates included: (1) Landcover classes (surface water bodies, sandy banks, grassland, brush thicket, seasonal dry forest, evergreen forest) classified from Landsat 8 Level 2 [84,85] imagery from the summer of 2019; (2) A simplified coverage of forest and matrix landcover classes reclassified from the general landcover classes variable; (3) measures of forest cover area (m^2^) generated using the landscapemetrics v1.5.4} package in R v4.1.3 [86]; (4) changes in forest cover from 2000 to 2021 [87]; (5) Euclidean distance to water basins (m); (6) elevation [88,89] (m); and (7) slope (%). We also used Landsat 8 Level 2 imagery to calculate surface reflectance-derived spectral indices of vegetation and environmental moisture quality [85], including: (8) Normalized Difference Vegetation Index [90] (NDVI); (9) Normalized Difference Moisture Index [91] (NDMI); and (10) Modified Normalized Difference Water Index [92] (MNDWI). Additional details about these 10 environmental covariates are included in Supplementary S1 Table. All 10 landscape covariates were resampled at a resolution of 30 m^2^ grid cell size to ensure matching spatial accuracy between data sources. Although climatic variables from datasets like WorldClim are commonly included in models of species distribution and niche divergence [76,82,93,94], the resolution of available datasets for this region were too coarse to include in our analysis. However, this exclusion of explicit climatic layers is unlikely to eliminate all climatic signals from our models, because climatic variation in this region covaries with environmental variables that we did include (e.g., elevation and forest cover), which can act as partial proxies for broad-scale climatic gradients [95].

For both species, we considered the same candidate environmental variables in each part of our analysis. This decision reflects the findings of previous research showing that variation in the niche requirements of *Eulemur* species is generally subtle, involving differences with the same broad environmental attributes rather than entirely distinct ecological preferences [67,96,97]. Moreover, because our goal was to test whether differences in niche associations across fragmented landscapes help explain variation in extinction risk, using the same set of variables allowed us directly to compare how each species responds to some of the main habitat conditions that are characteristic of the dry-deciduous forests they occupy in sympatry. We also note that for the purpose of this study we only included broad-scale environmental measures of landscape pattern and landcover types. As such, we ignore the role that finer-scale dynamic variables such as competition or the spatial distribution of resources (e.g., fruits) can have on the distribution and niche divergence patterns of *E. fulvus* and *E. mongoz*. Such variables are not included in this analysis due to difficulties associated with their spatial assessment at broad, landscape scales [98,99]. Thus, in this study we focus on the characterisation of Grinnellian niche of these two species as defined by their broad-scale habitat requirements, rather than their fundamental Eltonian niche that encompasses their functional role in their ecosystem (i.e., trophic position or foraging activities) [10,99].

### 2.4. Analysis

#### 2.4.1. Species distribution model.

We developed SDMs using MaxEnt v3.4.4 [81,100,101] and the *SDMtoolbox 2.0* [102] extension for ArcMap v10.8.1 [103] to estimate the influence of landscape composition and quality across geographic-space (G-space) on the occurrence of *E. fulvus* and *E. mongoz* across the ANP and MCF forest landscapes. MaxEnt is a widely used presence-only species distribution modelling approach that estimates the relative suitability across a study region of interest from occurrence records and background environmental predictors. MaxEnt contrasts environmental conditions at occurrence locations with those available across other background locations that are sampled from the broader biogeographic region [81,100,104]. Here, the relationship in observed environmental attributes between background and sampled occurrence locations were modelled using different candidate feature classes (e.g., linear, quadrative, etc.) for each covariate. The choice of feature class applied to the model depends on our ecological understanding of the species interactions with its environment as well as the characteristics of the available data (e.g., number of observations or the quality of interactions between covariates) [80,100]. For this reason, we investigated the impact of different feature types and their combinations on model performance, aiming to identify the most suitable type of feature class for each distribution model [104,105].

Thus, MaxEnt determines where a species is most probably present based on how similar the attributes of any one location in the area of study are to the average value of environmental attributes observed across all locations where the species is observed to be present – the more different a location is environmentally, then, the less probable it is that they are present there [80]. Importantly, the species distribution outputs generated by MaxEnt follow a generative approach where the probability of occurrence is jointly dependent on the distribution of the occurrence records and the background environmental information included in the model. This generative approach is beneficial to MaxEnt’s performance when the number of occurrence locations are low (minimum = 5); even when the number of input variables is greater than the number of sample observations [80,100,106] – although inference under very small sample sizes should be made cautiously [107]. However, these characteristics also mean that MaxEnt can be prone to overfitting when models are overly complex or when predictors are redundant, particularly in the presence of multicollinearity. For this reason, MaxEnt applies LASSO regularization (L1), which limits model complexity and improve general fit by imposing a penalties to feature weights on the model for each environmental covariate that is included. This penalty not only limits overfitting but also mitigates the effect of multicollinearity among predictors by reducing the contribution of highly correlated or less informative variables towards zero, effectively removing them from the model when they provide negligeable independent information [80]. Importantly, when predictors share environmental signal (e.g., correlated variables), regularization does not eliminate the underlying information content; rather, it tends to concentrate that shared signal into the predictor(s) and feature transformations that best improve model fit, while shrinking alternative, redundant representations toward zero [80,108,109]. Thus, the geographic range of the environmental gradient represented by a correlated pair can still be retained in the final model, even after the contributions of one member of the pair is reduced to limit redundancy.

The overall weight of the penalty is controlled using a constant regularization multiplier, where higher values reduce overfitting and autocorrelation in the probability output but may run the risk of loss in discriminatory ability if too large [80,100,105]. Because the L1 regularization multiplier influences both model generalization and the permutation importance of predictors, it is often necessary to examine a range of different regularization multipliers and their effect on model performance when validating model fit [104,105]. L1 regularization ensures that the choice of covariates included in the model can be guided by our ecological knowledge of the species of interest without concern about issues of autocorrelation or overfitting [80,104,106].

One of the main assumptions of MaxEnt is that sampling of species occurrence is random, which is not always possible due to common logistics and limitations of the sampling process [80,110]. To address possible issues in our data caused by sampling bias, we first used a spatial rarefication protocol whereby spatially autocorrelated observations were removed from the dataset by identifying spatial clusters of occurrence records within a neighbourhood distance of 30 m. We selected this neighborhood distance because it matches the pixel resolution of our environmental rasters, ensuring that no two occurrence locations fell within the same pixel. This approach preserves independence among presences and reduces spatial autocorrelation, which helps avoid overfitting and inflated suitability estimates in our two SDMs while maintaining the largest feasible sample size for the small *E. mongoz* dataset [111]. Next, we generated bias files of the sampling process for each species at each study landscape. This was done by delineating the sampled area using a buffered local adaptive convex-hull (LoCoH) polygon built from occurrence locations. We set the LoCoH parameter to α = 6, which maintained continuity among locally surveyed sites without forcing a single polygon to span our two landscapes, and we applied a 500 m buffer to reflect the common ranging distances reported for our two study species in comparable landscapes [65]. The bias file was used to restrict MaxEnt background selection to the surveyed region, thereby avoiding background draws from unsampled areas. This approach was combined with our rarefaction protocol to reduce within-survey clustering and limit the influence of locally overlapping observations in the model training process.

We developed and assessed MaxEnt models for *E. fulvus* and *E. mongoz*. For each of these models, we used the combined set of occurrence records collected between 2018 and 2020 and the 10 background covariates discussed in section 2.3. The lemur dataset included 353 occurrence records for both *Eulemur* species in ANP and MCF. Of these, 266 records were of *E. fulvus*, and 77 records were of *E. mongoz*. After spatial rarefication, 285 records were retained: 229 of *E. fulvus* and 56 of *E. mongoz*. In the case of *E. fulvus*, the distribution of records across each landscape was somewhat consistent, with slightly more observations at ANP (N = 128) than at MCF (N = 101). In contrast, the distribution of occurrence records for *E. mongoz* at each landscape was highly skewed. The majority of records were collected from ANP (N = 48), while only a few records were collected at MCF (N = 8).

Because these covariates exhibit high autocorrelation (S2 Table), we evaluated model fit across a range of L1 regularization multipliers to identify settings the best account for variable redundancy while minimizing overfitting. To this end, we used the spatial jackknife protocol for training and validation of our MaxEnt models available on the *SDMtoolbox 2.0* extension [102]. The spatial Jackknife protocol applies a geographically structured k-fold cross-validation [105,112] replication procedure that trains and evaluates each of our species models using different parameters that are known to impact model fit. This evaluation is completed across three spatially segregated locations within our training region, which are defined by a cluster of equal numbers of sampled occurrence records [105]. Training runs were parametrized using varying permutations of the L1 regularization multiplier and feature class combinations while using a randomized subset containing 20% of the sampled occurrence records (*E. fulvus* n = 45; *E. mongoz* n = 11) and the background environmental data on two out of the three training locations for every run. Model fit was then evaluated using the remaining 80% of occurrence locations and background data from the withheld subset. This process was repeated for every permutation of *n-1* spatial clusters, multiplier setting, and feature class combinations (Supplementary S3 Table for a list of the top five SDMs evaluated for each species).

The best model was decided by first comparing test of omission rates (OR) and then estimates of the area under the curve (AUC) from each candidate run – selecting the models with the lowest OR and highest AUC. If the top training models exhibited comparable metrics, then the model with the simplest feature class was selected. OR tests for each fold were calculated through binary comparisons of the number held-off occurrence locations incorrectly predicted as not suitable by a 10-percentile training presence cut-off classification of the continuous probability distribution [100,102,112]. Low OR values are indicative of lower overfitting to the training data compared with other similar models. AUC was estimated as a threshold-independent measure of the model’s discriminatory ability to differentiate between locations where the species was observed to be present and randomly chosen areas where the species was predicted as absent [100]. Higher AUC values here indicate a more generalized predictive capacity. For interpretation purposes, we used the values from Hosmer et al. [113] – where AUC values between 0.7-0.8 were acceptable, 0.8-0.9 were excellent, and >0.9 were exceptional. After all parametrized runs were completed and evaluated, the final model of each species was run using the optimal parameters on the complete set of observations and background covariates plotted across the entire biogeographic region of interest.

Final models for each species were developed using K-Fold Cross-Validation. This replication procedure divides the data across five replicate folds, holding-off a randomized 20% of occurrence locations each run as a validation set for model testing while the remaining folds serve as the training data. This process was repeated across 100 replicate runs and AUC metrics were computed to calculate discriminating power [81,100]. The output, a continuous probability distribution that predicts where each species was most likely to be present, was generated by averaging the output of each replicate run [104]. This continuous probability distribution is a post-transformation of the raw, cumulative MaxEnt output that was developed using a Bernoulli generalized linear model with a ClogLog link function [80,81]. A ClogLog link function was used because it does a better job modelling the top-range of the distribution than a comparable logit link function – thus, it helps to better highlight the locations of preferred habitat sections [81]. To represent areas where each of our two study species are likely present (suitable habitat = 1) and absent (nonsuitable habitat = 0), we reclassified the continuous probability distribution estimates into binary distribution estimates. This was done using the 10-percentile logistic threshold (10PLT), which uses 90% of occurrence records and background information to define suitable habitats. The 10PLT is particularly useful for datasets where some degree of error is likely to be present due to sampling bias, as it assumes a balance between underrepresentation in some areas of the study region and overrepresentation in others. This conservative threshold is also is also well suited for conservation applications because it is less likely to classify areas as suitable where the species is unlikely to occur [76,100].

In addition to the omission rate and full AUC metrics, we evaluated the final models using a partial receiver operating characteristic (ROC) approach that is well suited to evaluating the predictive outputs of ENMs by focusing on evaluating the biologically relevant, low omission portion of ROC space, and avoids well-known limitations of traditional full AUC comparisons [114]. We implemented partial ROC in R v4.1.3 [86] using the fpROC v0.1.0 [115] package in in R v4.1.3 [86], which employs Peterson et al.’s [114] protocol by evaluating model predictions within each species’ accessible area (M), masking the probability distribution of each species’ final model to the same background region used in model calibration. We then assigned an omission tolerance of E = 5%, so performance was assessed over a high sensitivity region, using 1000 bootstrap iterations with a 50% subsampling of withheld test occurrences per iteration. Occurrence data were partitioned using a reproducible random 80/20 train–test split; evaluation used 45 test points for *E. fulvus* and 11 test points for *E. mongoz*. Statistical significance was assessed as the proportion of bootstrap replicates with AUC ratios ≤ (with a conservative +1 correction). To ensure robustness in the *E. mongoz* model given the small test sample size of 11 observations, we repeated the analysis using five different random seeds and obtained no meaningful differences in the estimated AUC ratios.

We evaluated the area amount (ha) of suitable habitat and its spatial aggregation (contagion) for the binary distribution estimates of *E. fulvus* and *E. mongoz* using the landscapemetrics v1.5.4 [116] package. Moreover, we compared differences in the binary distribution estimates of each species using a conditional spatial overlay which allowed us to map areas of MCF and ANP uniquely suitable to each species, as well as areas of sympatry commonly suitable for both. For each species model, we also generated partial response curves (PRCs) and a summary of the relative permutation importance of each environmental covariate. PRCs show how the predicted probability of occurrence changes because of variation in each environmental covariate, while keeping all other covariates at their mean sampled value. Finally, estimates of permutation importance for each covariate quantifies how much model performance declines after that variable’s values are randomly shuffled among presence and background locations, thereby breaking its association with occurrence; larger declines indicate greater reliance on the variable in the fitted model [80]. In this way, permutation importance summarizes each variable’s influence on the final predicted probability distribution, although interpretation should be cautious when predictors are correlated because shared environmental signal may be represented by only a subset of retained covariates.

#### 2.4.2. Environmental niche comparison.

To examine the environmental drivers of *E. fulvus* and *E. mongoz* occurrence across MCF and ANP, we examined their patterns of ecological niche overlap and divergence using an environmental-space (E-space) approach for niche similarity analysis. Assessments of niche similarity were completed using the Humboldt v1.0.0.0420212 [82] package in R, which uses methods first proposed by Broennimann et al. [117] and Qiao et al. [118] that operate under the assumption that the contemporary distributions of most species are in states of non-equilibrium. Under this assumption of non-equilibrium, species distributions are predicted to reflect a truncated manifestation of a species’ environmental niche requirements (E-space) across their geographic range (G-space), rather than their fundamental niche requirements. With this niche equivalence approach, we can model patterns of niche overlap and divergence under non-equilibrium states for *E. fulvus* and *E. mongoz* by explicitly incorporating information about the degree of similarity of analogous environmental conditions that were accessible to each species within their sampled range of occurrence [82].

Estimates of niche patterns for either species were developed using their rarefied occurrence datasets and a subset of the 10 environmental covariates. As recommended by Brown and Carnaval [82], the choice of environmental covariates added to the niche representation of our two species was determined using the *humboldt.top.env()* function. This function runs a generalized boosted regression model (GBRM) [119] to quantify the importance of each covariate to estimates of either species’ distribution. We selected a minimum threshold of 5% contribution to the GBRM to determine the subset of covariates that we would include in the niche similarity analysis. To facilitate multivariate comparison of environmental conditions with different scales of variance, all 10 covariates were scaled to a mean of 0 and a standard deviation of 1 prior to reduction and analysis. Furthermore, we trimmed the geographic extent of environmental information of each species using a buffered local adaptive convex-hull (LoCoH) polygon that mapped the maximum extent of observed localities with a buffer distance of 500 m around its periphery. Buffered LoCoHs were generated from the pool of occurrence records of either species. We trimmed our covariates to this extent to ensure that niche estimates of either species only included information on environmental conditions for locations in the accessible range of either species’ observed points of occurrence.

We quantified environmental niches patterns of *E. fulvus* and *E. mongoz* using 2-dimensional principal component (PC) analysis where E-space was characterized by the first two components of the scaled environmental covariates retained for analysis [82]. For each species, we developed kernel density isopleths mapped onto the first and second components to create continuous estimates of each species’ E-space distribution. Kernel density isopleths for each species were developed from (1) all locations within their sampled geographic range and (2) the specific locations where they were observed, mapping the range of the environmental conditions used by each species as well as their distribution of occurrence in this E-space [82,117]. Because strong correlations between the environments occupied by each species can bias estimated of their occupied E-space, kernel density isopleths were corrected by the abundance of different environmental conditions present [82]. Differences between the occupied E-space of each species and their respective environmental E-space were then quantified using equivalence and background statistics.

We compared the degree of overlap and divergence between niche estimates of *E. fulvus* and *E. mongoz* by computing Schoener’s niche identity (D) and Warren’s niche background (I) [82,120] estimates. Both of these metrics output niche similarity estimates, where a value of 0 indicates no overlap in environmental preference and a value of 1 indicates identical environmental preference [120]. We assessed the significance of niche similarity estimates by computing a one-tailed niche equivalence test, which examined whether the observed niche overlap between both species was greater than their respective overlap against null background distributions. The null background distributions were generated by pooling occurrence points from both species and then randomly resampling them into one of two background groups – where the number of occurrences in each group matched the number of occurrences sampled for either species. This process was repeated for 1,000 iterations, estimating Schoener’s D and Warren’s I measures between the two groups at each step. Next, we evaluated the ability for the equivalence statistical tests to detect differences in the E-space available to each species. This background statistical test examines whether the E-space distributions of the two species differ more than expected, given the range of environmental conditions across their region of occurrence. This process was done by comparing the niche similarity (Schoener’s D and Warren’s I) estimates of *E. fulvus* and *E. mongoz* to overlap in the similarity estimates of one species and the random shifting of the spatial distribution of the second species across their sampled range of occurrence. This shifting process was replicated 1,000 times or until all sampled locations for the second species had shifted into novel environments available within the range of their G-space while maintaining the general spatial structure of sampled locations. This replication process was completed to generate a null distribution of E-space available in the geographic range of the second species that could be used to quantify the impact that shifts in their distribution have on estimates of their occupied E-space [82].

Equivalence and background tests for the niche similarity patterns of *E. fulvus* and *E. mongoz* were first competed using the entire E-space distribution of both species to complete an analysis of their patterns of overlap (Niche Overlap Test – NOT). To examine patterns of divergence (Niche Divergence Test – NDT), the equivalence and background tests were completed by only using analogous portions of their respective E-space where the two species overlapped. Significance level for the equivalence and background statistical tests was set at α = 0.05. We used the key presented by Brown and Carnaval [82] to interpret the ecological relevance of significant equivalence or background tests when comparing their NOT and NDT parametrizations (See Supplementary S5 Table for a key listing possible ecological interpretations of NOT and NDT comparisons).

## 3. Results

### 3.1. Species distribution models

MaxEnt models for each species showed strong discriminatory power on held out folds based on their AUC and mean omission rate (OR) estimates. With a mean cross-validated area under the curve (AUC) value of 0.73 for *E. fulvus* (S2 Fig – A) and an AUC value of 0.89 for *E. mongoz* (S2 Fig – B). The AUC values from each MaxEnt model suggest acceptable performance by the *E. fulvus* model and excellent performance by the *E. mongoz* model. Our *E. fulvus* model also exhibits high convergence between the mean OR on held out folds and the predicted OR. This species-model was further supported by a significant binomial test of OR (average test OR = 0.11; *P* < 0.0001). With regards to the *E. mongoz* MaxEnt model, there is some small degree of deviance between the OR on held out folds and the predicted OR at lower to middle cumulative thresholds. Nevertheless, the model is supported by a significant binomial test of OR (average test OR = 0.16; *P* = 0.0002).

Partial ROC evaluation indicated that MaxEnt models for both species performed significantly better than random expectations in the low-omission region (E = 5%; Table 1).The *E. fulvus* model yielded a mean partial AUC ratio of 1.166 (95% bootstrap CI = 1.130–1.316; n = 45 test points), whereas for the *E. mongoz* model, the partial AUC ratio is 1.723 (95% bootstrap CI = 1.688–1.819; n = 11 test points). For both species, no bootstrap replicate produced an AUC ratio ≤ 1 (naïve p = 0). Applying a conservative +1 correction gave a p < 0.001 for each model, providing strong evidence of accurate predictive performance in comparison to random expectations under the partial ROC test. Repeating the *E. mongoz* analysis across five different random seeds produced consistent ratio estimates (S4 Table), indicating that results were not sensitive to the random split of occurrence due to the small size of the sample.

**Table 1:** Partial ROC evaluation summary for final E. fulvus and E. mongoz MaxEnt models within accessible area M. Partial ROC ratio and 95% bootstrap CI focus on the high-sensitivity (low-omission) region defined by E and summarizes performance as an AUC ratio. Also present is a summary of the parameters for the evaluation and an estimate of the full AUC value for each species’ model.

Species	n Test Points	Omission Tolerance (E %)	Bootstrap Iterations	Full AUC	Mean partial AUC ratio	95% bootstrap CI	P-Value
*E. fulvus*	45	5%	1,000	0.73	1.166	1.130–1.316	p < 0.001
*E. mongoz*	11	5%	1.000	0.89	1.723	1.688–1.819	p < 0.001

**Fig 2:**
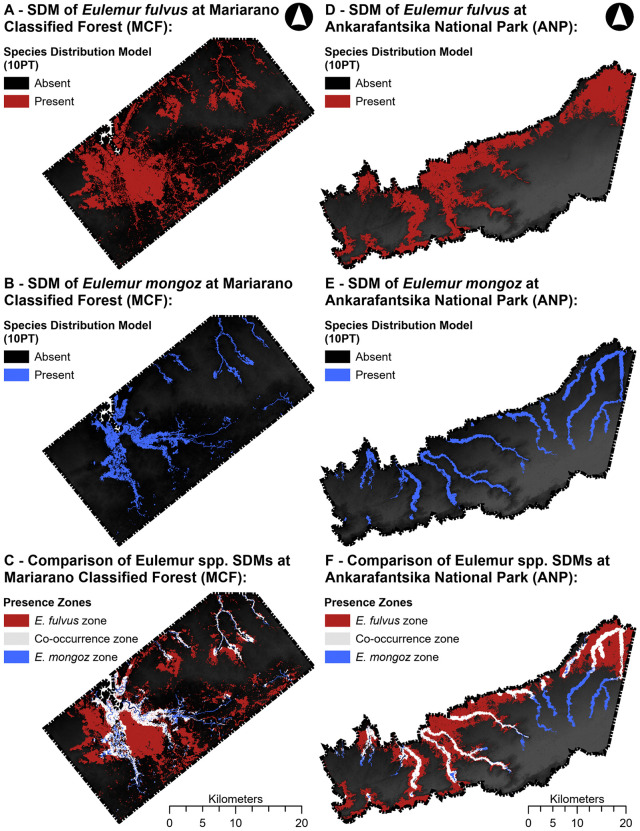
Probability distribution of E. fulvus (red) and E. mongoz (blue) across ANP (A, B) and MCF (D, E). Also present is the comparison of their distribution patterns in each landscape, marking zone of occurrence unique to each species (EF – red and EM – blue) as well as their zone of co-occurrence (white). Basemap hillshade (greyscale) derived from the Shuttle Radar Topography Mission (SRTM) DEM (U.S. Geological Survey/NASA). Protected-area boundary for ANP digitized by the authors from an official Madagascar National Parks map provided during fieldwork (georeferenced using field control points); MCF boundary delineated by the authors to encompass surveyed forest patches. MaxEnt model outputs (10-percentile training presence) are original to this study.

SDMs for both species predicts a heterogenous probability of occurrence across ANP and MFC, in association with variation in environmental conditions (Fig 2). For *E. fulvus*, the predicted probability distribution covers an area of 48,591.72 ha and exhibits low zonal aggregation (Contagion = 44.26) indicative of a more continuous coverage (Fig 2: A & B; Table 2). For *E. mongoz*, the predicted probability distribution is much smaller than *E. fulvus*, covering an area of 17,757.00 ha and exhibiting higher zonal aggregation (70.06) (Fig 2: C & D; Table 2). Visual inspection of the probability distribution outputs of each species indicates that the distribution of *E. fulvus* closely follows the distribution of forest vegetation at ANP and MCF (Fig 1–2). In contrast, the distribution of *E. mongoz* appears to be constrained to the watershed basins in each landscape. This pattern is most apparent at ANP, where the predicted distribution of *E. mongoz* closely follows the river basins of the valleys. Comparing overlap between the probability distribution output of each species model, the predicted zone of co-occurrence for both species covers an area of 12,735.99 ha (Fig 2: E & F; Table 2). Furthermore, the exclusive zones for *E. fulvus* (area = 35,855.73 ha) covers an area seven times larger than that of *E. mongoz* (area = 5,021.01 ha) (Table 2).

**Table 2:** (A) Estimates of the total occurrence area (ha) and zonal aggregation (Contagion) based on the classified output of our species distribution models for sympatric E. fulvus and E. mongoz populations. (B) Estimates of the total area (ha) for zones where only ones of the E. mongoz and E. fulvus populations are predicted, as well as their predicted zones of co-occurrence.

Species	Area (ha)	Contagion
**(A)Species-specific SDM Outputs**		
*E. fulvus*	48,591.72	44.26
*E. mongoz*	17,757.00	70.06
**(B)Species-Overlap of SDM Outputs**		
*E. fulvus* zone	35,855.73	NA
*E. mongoz* zone	5,021.01	NA
Co-occurrence zone	12,735.99	NA

Estimates of relative permutation importance for each covariate show that probability distributions for each species are driven by different combinations of environmental attributes (Table 3). For *E. fulvus*, variation in elevation from sea level (m) across ANP and MCF (% importance = 52.6) provided the most useful training information of all 10-input covariate. Less but otherwise important covariates included changes in forest cover between 2000 and 2021 (% impt = 22.2), amount of forest cover (ha) (% impt = 14.7), the quality of vegetation (Normalized Difference Vegetation Index – NDVI) (% impt = 6.5), and the distance to water basins (m) (% impt = 2.8). Covariates such as the content of vegetation moisture (Normalized Difference Moisture Index – NDMI) (% impt = 0.5), the percentage of slope rise (% impt = 0.3), the distribution of surface water bodies (Modified Normalized Difference Water Index – MNDWI) (% impt = 0.2), the type of habitat cover type (% impt = 0.2), and the distribution of forest cover (% impt = 0.0) had minimal to no importance to the probability distribution estimate of *E. fulvus*. With regards to *E. mongoz*, their distance to water basins (m) (% impt = 87.7) provided the most important training information, followed by the content of vegetation moisture (NDMI) (% impt = 9.8), and the amount of forest cover (ha) (% impt = 1.1). The remaining seven environmental covariates had minimal to no importance to the probability distribution of this species (Table 3).

**Table 3:** Estimates of the relative permutation importance of 10 independent environmental covariates to our species distribution models for sympatric E. mongoz and E. fulvus populations.

Covariate Name	*E. Fulvus*	*E. Mongoz*
Elevation (m)	52.6	0.0
Slope (%)	0.3	0.0
Habitat class	0.2	0.5
Forest coverage	0.0	0.9
Forest loss (2000–2021)	22.2	0.0
Forest area (ha)	14.7	1.1
NDVI	6.5	0.0
NDMI	0.5	9.8
MNDWI	0.2	0.1
Dist. to water basins (m)	2.8	87.7

**Fig 3:**
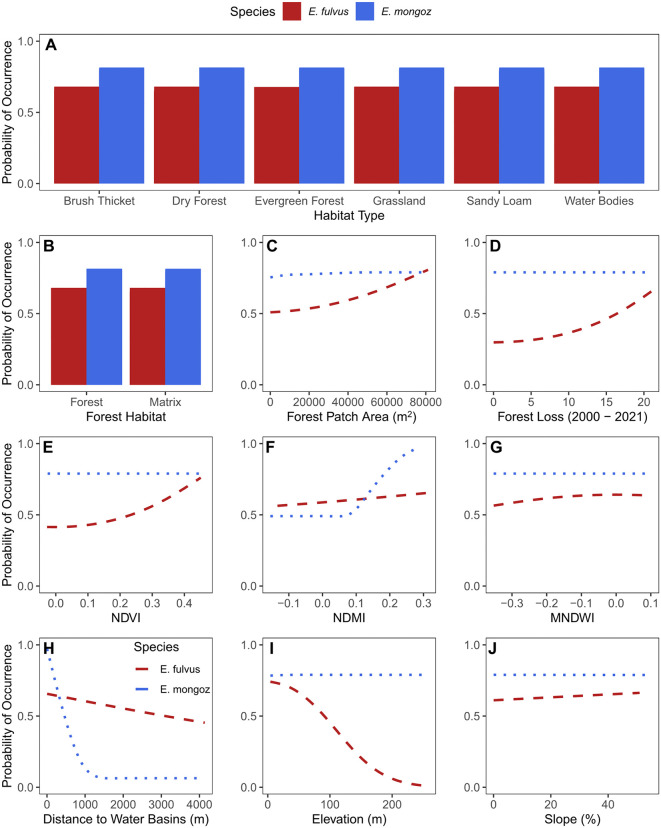
Partial response curves (PRCs) for the top E. fulvus and E. mongoz SDMs. PRCs show how the probability of occurrence of each species varies because of variation in each environmental covariate, while keeping all other covariates at their mean sampled value.

Partial response curves (PRCs) show the relationship between our candidate environmental covariates and the probability distribution of each species (Fig 3). In our *E. fulvus* distribution model, variation in the probability of occurrence across the two study landscapes was negatively associated with changes in elevation (m) – species occurrence was predicted to be higher at elevations closer to 0 m from sea level (Fig 3: I). Moreover, the model also predicted that *E. fulvus* occurrence was positively associated with variation in the temporal patterns of forest cover change (2000–2021), forest area (ha), and NDVI across ANP and MCF. These results indicate that *E. fulvus* groups are most likely present across large and intact lowland forests marked by high vegetation quality (Fig 3: C–E). Our model also predicts a negative association between the probability of occurrence of *E. fulvus* groups and their distance from water basins (m) (Fig 3: H). For *E. mongoz*, variation in the probability of occurrence was strongly associated with their distance from water basins (Fig 3: H). Furthermore, there was a positive association between their probability of occurrence and variation in the amount of vegetation moisture present and the area of forest patches (Fig 3: F, C). These results suggest that *E. mongoz* is most commonly present across larger forest patches that exhibit high moisture content due to their immediate proximity to water basins. Importantly, while the probability of occurrence of both species exhibits some degree of association with their distance from water basins, the geographic magnitude of this relationship is quite different. For *E. fulvus*, there is a gradual decrease in their probability of occurrence with increasing distance to water basins, yet the difference in probability between the highest and lowest extent is not greater than 16%. In contrast, for *E. mongoz* we predict a strong association where the probability of occurrence of this species reaches the lower margins of the distribution (i.e., expected absence) at locations that are over 1 km away from surface bodies of freshwater (Fig 3: I).

### 3.2. Assessment of ecological niche overlap and divergence

**Fig 4:**
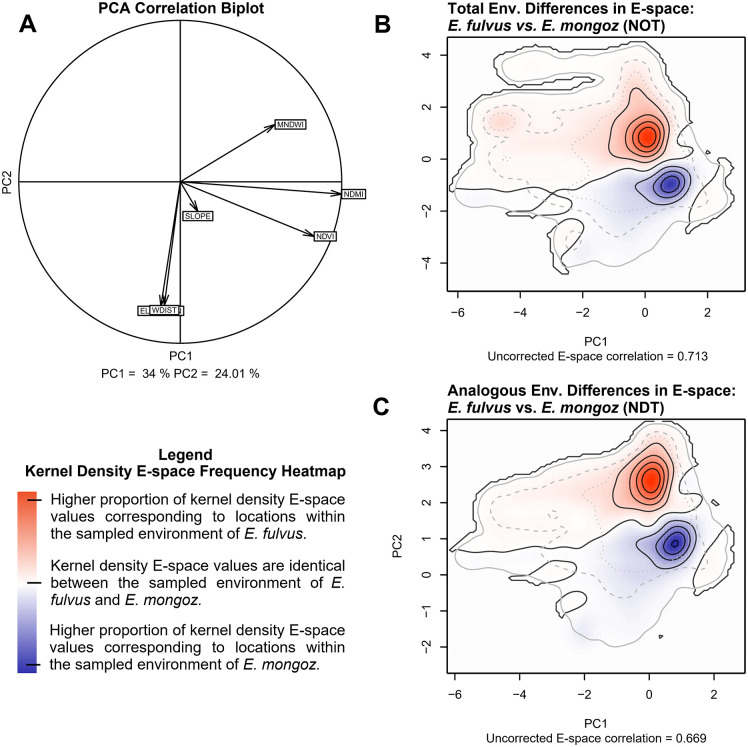
Niche comparison of the environmental conditions occupied by populations of E. fulvus and E. mongoz in northwestern Madagascar. Panel A includes the PC correlation biplot and the proportion of variance in the environmental data that can be presented in 2-dimensional E-space. The environmental covariates included in this portion of our analysis are presented inside the correlation biplot, projected onto the 2-dimensional E-space to represent their influence on the niche patterns of both species. The length of each arrow represents how well the covariate explains the distribution of the data. If two arrows point in the same direction, those covariates are correlated. Any two arrows pointing in orthogonal (90-degree angles) are unrelated to each other. Arrows pointing in opposite directions are negatively correlated. Panels B and C include kernel density isopleths mapping differences between the E-space of environmental conditions in the sampled range of E. fulvus and E. mongoz for our niche overlap (NOT) and niche divergence (NDT) tests respectively. Interpretation of the isopleths should follow the key listed in the legend. These two panels also list the amount of correlation present in the E-space of both species before correcting for the abundance of common environmental ranges.

The principal component (PC) comparison of niche similarity between *E. fulvus* and *E. mongoz* shows that 58.01% of the environmental space (E-space) variance (PC1 = 34% and PC2 = 24.01%) can be represented in a two-dimensional axis (Fig 4: A). The first component (PC1) is primarily driven by the positive association of covariates mapping environmental moisture and quality, as well as the distribution of surface waterbodies (i.e., NDMI, NDVI, and MNDWI). The second component (PC2) is driven by the negative association of covariates mapping topographic characteristics (i.e., elevation (m), distance to water basins (m), and slope (%). In the PC biplot (Fig 4: A), the NDMI and NDVI variables clustered closely together, contributing most to the environmental differences in the E-space distribution of each species. These variables are strongly positively correlated with MNDWI and slope (%), but less so with elevation (m) and distance to water basins (m). Elevation (m) and distance to water basins (m) cluster together in our analysis of both species’ E-space variance, correlating positively with slope (%), which had the least relevance to the E-space of *E. fulvus* and *E. mongoz*, and negatively with MNDWI.

**Fig 5:**
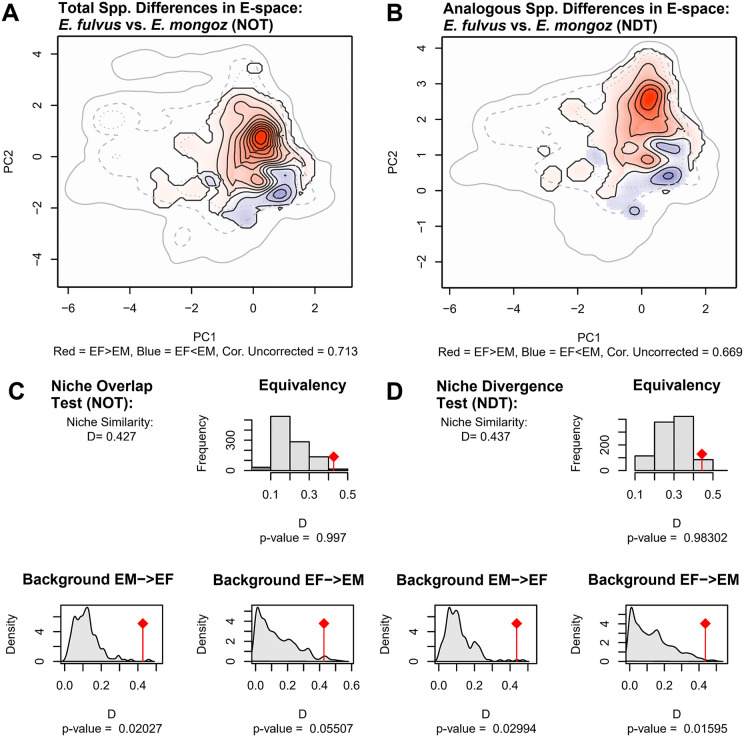
Comparison of niche equivalence and niche background tests between populations of E. fulvus and E. mongoz in northwestern Madagascar. Panels A and B visualize each species’ distribution across environmental space (E-space) using kernel density isopleths, thereby illustrating how each species’ E-space use clusters and overlaps relative to the other. Panel A shows kernel density isopleths used for the niche overlap test (NOT), and Panel B shows isopleths used for the niche divergence test (NDT), mapping differences between the E-space conditions where E. fulvus and E. mongoz where observed and indicating the correlation structure of the environmental variables before correction. Panels C and D present summaries of our niche overlap (NOT) and niche divergence (NDT) tests, including estimates of niche equivalence in the E-space of E. fulvus and E. mongoz as measured by Schoener’s D niche similarity index, as well as the results of our tests of niche equivalence and background comparison.

For our Niche Overlap Test (NOT), which compares the equivalence of niche patterns between *E. fulvus* and *E. mongoz* across the entire coverage of their respective E-space, we obtained a non-significant equivalence statistic (Schoener’s D = 0.427, p-value = 0.997). This estimate is indicative of a high degree of similarity in the niche patterns of both species across the total range of their respective E-space, which is further supported by the large area of the co-occurrence zone of both species mapped in our SDMs (Fig 2: C, F). Moreover, background statistical comparison for each species was significant when comparing the null distribution of available E-space in the habitat of *E. mongoz* against the observed E-space of *E. fulvus* (Background *EM* → *EF*: p-value < 0.02) but not when comparing the null distribution of available E-space for *E. fulvus* against the observed E-space of *E-mongoz* (Background *EF* → *EM*: p-value = 0.05), meaning that the niche patterns of *E. fulvus* and *E. mongoz* were more similar than expected by random chance since at least one comparison was significantly different (Fig 5: A, C). Restricting our analysis to the analogous E-space of *E. fulvus* and *E. mongoz* (Niche Divergence Test – NDT), we found similar results to the NOT analysis. We obtained a non-significant equivalence statistic (Schoener’s D = 0.437, p-value = 0.983) across the range of their analogous E-space. Background statistics were also significant when comparing the null distribution of available E-space in the habitat of one species against the observed E-space of the other species (Background *EM* → *EF*: p-value < 0.01; Background *EF* → *EM*: p-value < 0.05) (Fig 5: B, D). Based on the interpretation key presented by Brown and Carnaval (S5 Table), the non-significant results of the equivalence statistic and significant results in the background statistics for the NOT and NDT comparisons suggest that there is strong evidence that the niche requirements of both species are equivalent across E-space.

Differences in the kernel density isopleths of available E-space for *E. fulvus* and *E. mongoz* support the results of the equivalence and background statistics (Fig 4: B, C: Fig 5: A, B; See also S3 Fig). In the NOT and NDT comparisons, kernel density isopleths exhibit a high range of E-space overlap between the two species, especially along PC1. Available E-space in the habitats of *E. fulvus* and *E. mongoz* is positively correlated with PC1 indicating that both species likely exhibit similar preference for vegetation conditions and the availability of water in their environment. Despite this significant range of overlap, the E-space of each species shows key differences. Kernel density estimates show that available E-space in the habitat of *E. fulvus* is more abundant in the upper range of PC2, whereas available E-space for *E. mongoz* is most abundant in the lower range of PC2. Differences in the E-space distribution of each species along PC2 likely indicate the *E. mongoz* were most commonly present at lower elevations and in close proximity to water basins than *E. fulvus* – similar to the results of our SDM analysis.

## 4. Discussion

The results of our analysis offer partial support for the hypotheses proposed in this study. In geographic space, the predicted distribution patterns of *E. fulvus* and *E. mongoz* differ markedly, aligning with our first hypothesis. The SDM for the Vulnerable [58] *E. fulvus* predicts a relatively broad and continuous range across both MCF and ANP. This distribution closely follows gradients in elevation, forest cover, and vegetation quality, suggesting that *E. fulvus* is able to persist across a wide array of habitat conditions. In comparison, the predicted distribution of Critically Endangered [59] *E. mongoz* is considerably smaller and more fragmented. Its occurrence is largely restricted to areas near water basins and forest patches with high vegetation moisture, indicating a narrower set of environmental tolerances. These spatial patterns are consistent with the idea that broader niche requirements confer greater ecological flexibility, allowing *E. fulvus* to maintain a more extensive and connected distribution range in fragmented landscapes than *E. mongoz*.

However, our second hypothesis, where we predict that *E. fulvus* would occupy a broader environmental space than *E. mongoz*, was not supported. Despite differences in their predicted geographic distributions, the two species exhibited substantial overlap in the range of environmental conditions they occupy. While kernel density isopleths revealed some differentiation along topographic gradients (particularly in the second principal component of E-space), the results of the NOT and NDT comparisons did not indicate significant divergence in environmental niche breadth. This overlap is further reflected in the spatial co-occurrence of *E. mongoz* niche space within portions of *E. fulvus*’ predicted range. These findings suggest that, although *E. mongoz* is more spatially restricted, it does not necessarily occupy a distinct or more specialized niche in terms of the environmental variables considered. Instead, its limited distribution may reflect other ecological constraints – such as behavioral or demographic sensitivities to topographic and vegetation patterns as well as long-term patterns of forest habitat loss – that restrict their use of preferred habitats but are not fully captured by the environmental covariates used in our models.

### 4.1. Variation in distribution patterns

The differences in the size and continuity of the predicted distributions of *E. fulvus* and *E. mongoz* highlight important contrasts in the flexibility of their habitat preferences. While the predicted distribution of *E. mongoz* is highly fragmented, restricted to lowland forests in zones of moist vegetation near freshwater basins, *E. fulvus* exhibits a broader and more continuous distribution across the study landscapes. This distribution pattern aligns with low elevation areas where continuous forest cover is dense and vegetation quality is highest, suggesting that *E. fulvus* is better capable of using the available forest habitats in ANP and MCF.

Our top-performing SDM indicates that the probability of *E. fulvus* occurrence is primarily shaped by topographic variation and forest structure – particularly canopy cover, patch area, and vegetation quality – across both ANP and MCF. In seasonal dry-deciduous forest landscapes, topographical changes in elevation strongly influence local hydrology, which in turn affects the distribution of freshwater sources and the quality of forest patches [121–125]. As a result, *E fulvus* groups may be more frequently associated with lowland forest areas where water availability and continuous forest cover are more prevalent [64,126–128]. The importance of elevation patterns across MCF and ANP for the distribution of *E. fulvus* may thus reflect their preference for larger patches of forest habitats that are characterized by close canopy cover and high vegetation activity, which in northwestern Madagascar are most commonly located along lowland topographic positions. These findings suggest that in these landscapes, *E. fulvus* primarily requires access to sufficiently large forest habitats. While its likelihood of occurrence may fluctuate with changes in vegetation quality or disturbance levels, such variation does not appear to exclude the species from inhabiting these areas.

Our results are consistent with previous studies on the behavioural and population ecology of *E. fulvus* and other *Eulemur* species [55,60]. Specifically, these results reinforce the importance of forest patch attributes in shaping occurrence patterns. In particular, the patterns we observed emphasize the influence of forest patch size and, relatedly, patch quality and connectivity for determining the distribution of *E. fulvus* and its congeners. For instance, in the Ambanjabe Forest Fragment Site (AFFS), located in the western portion of ANP, the probability of occurrence of *E. fulvus* has been predicted to vary in association with the size and connectivity of forest patches [55,60]. In this landscape, *E. fulvus* is more likely to occur in large, well-connected patches that are capable of supporting its relatively large home ranges and group sizes [23,55]. Comparable relationships between habitat extent/connectivity and distribution have also been observed in other *Eulemur* species, suggesting that variation in forest patch size and connectivity are among most influential habitat attributes that affect where lemurs are most commonly present – particularly for taxa with large home ranges and population sizes [52,129–132].

While our analysis evaluates distribution patterns in relation to topographic and vegetation features, it does not directly test the behavioural and demographic mechanisms that enables populations of *Eulemur* to persist while their habitat patches are being lost, fragmented, or degraded. Nonetheless, prior research outlines plausible mechanistic context to interpret why habitat size, connectivity, and quality emerge as strong correlates of occurrence for these species [52]. Many *Eulemur* species – including *E. fulvus* – exhibit behavioural flexibility at the individual and population levels that allows them to cope with short-term changes in habitat characteristics. Examples include behavioural strategies like shifting activity budgets and flexible foraging preferences [66]. These traits enable some *Eulemur* species to optimize their resource access during periods of scarcity, as seen with species like *E. coronatus* [133], *E. collaris* [131,134,135], *E. flavifrons* [136], *E. rufifrons* [127,137–139], and *E. fulvus* [69,75,140]. At the individual level, power-feeding energy-maximizing strategies allow increasing energy foraging effort to search and acquire patchily distributed resources at the expense of resting time [66,141]. At the Ampijoroa Forest Station in ANP, for example, Sato [74,75] reported that during the dry season, *E. fulvus* adjusts diet composition and extend foraging time when switching to fall back foods. Similar seasonal strategies have been documented at the Tsinjoarivo‐Ambalaomby New Protected Area in Eastern Madagascar, where *E. fulvus* groups maximize foraging time during seasonal lean periods [140].

At broader scales, some *Eulemur* species may also exhibit flexibility in home range size, group composition, and density in response to habitat conditions [52,67,142]. In ANP, *E. fulvus* groups shift home range use in response to seasonal variation in fruit availability [69], indicating that ranging behaviour can track temporal resource variation. However, direct evidence for demographic plasticity in *E. fulvus* (e.g., systematic shifts in group size or density with fragmentation) remains limited. In closely related species with similar social characteristics (i.e., large, multi-male multi female groups), such as *E. rufifrons* and *E. collaris*, group size and density vary across habitat edges and degraded areas. For instance, *E. rufifrons* occurs at lower densities in edge-zones where preferred food types are less common [137], and its home range and group size vary with seasonal food and water availability [126,139,143]. Likewise, *E. collaris* group size and home range patterns shift with the degree of environmental degradation, such that groups are usually smaller and range over larger distances in degraded habitats [131,134]. Collectively, these findings suggest that group-living *Eulemur* species can adjust their social and spatial organization to cope with environmental stressors [67,68,144,145]. Given the ecological similarities among these taxa, such mechanisms provide a plausible context for our observed association between *E. fulvus* patterns of occurrence and forest patch characteristics. However, further research is still needed to confirm whether *E. fulvus* exhibits comparable, systematic variation in group dynamics and population density across fragmented landscapes.

The predicted distribution of *E. mongoz* across ANP and MCF indicates that occurrence is tightly constrained to moist forest habitats near water basins. This pattern aligns with previous findings that *E. mongoz* populations in Madagascar are typically found in intact, lowland dry forests characterized by dense, high-quality vegetation and minimal anthropogenic disturbance [73,76]. In our models, the strongest spatial signal is tied to topographic variation – specifically, proximity to water basins at low elevations – similar to occurrence patterns of *E. fulvus* and other congeners [106,126,127]. Proximity to water basins is important because is likely captures the distribution of persistent microhabitat conditions along lowland areas, tend to have deeper soils and higher water tables, which offer a buffer against seasonal moisture fluctuations and promote more stable food and water availability between the dry and wet seasons [146–148]. Notably, this topography–occurrence association is more pronounced for *E. mongoz* than for *E. fulvus*. While *E. fulvus* can tolerate a broader range of vegetation conditions within continuous forests, potentially reflecting flexible foraging and ranging strategies [66,69,75], the predicted distribution of *E. mongoz* is concentrated to microhabitat conditions characterized by higher vegetation moisture and greenness, as well as close proximity to freshwater.

Our interpretation of the predicted distribution patterns of *E. mongoz* should be considered in light of the limitations discussed earlier; that is, our analysis identifies environmental correlates of predicted occurrence rather than directly establishing behavioural or demographic mechanisms influencing this pattern, such as their diet, territoriality, or social organization. Nevertheless, the broader ecological literature offers a possible explanation for the pronounced spatial constraint that we detect – an interaction between their dietary preferences and their pair-living, territorial social system. In dry forest landscapes, lowland habitats near water basins experience less seasonal variation in vegetation activity and moisture, helping maintain the availability of preferred food resources between seasonal periods [121,125,146,147,149]. *E. mongoz* is strongly frugivorous, with a diet that favors fruits, flowers, and young leaves [66]; and similar to other *Eulemur* species, it exhibits a diet with flexible composition that changes across seasonal periods [65,66,150]. However, unlike other conspecifics, its preferred resources include a greater proportion of seasonally variable items (i.e., fruit and flower nectar) compared less variable resources like leaf foliage [61,66,150] – which can increase dependence for lowland microhabitats that buffer them against dry conditions.

A further constraint is likely imposed by space use and territoriality. Unlike congeners such as *E. fulvus* that can shift home range use in response to resource availability, *E. mongoz* lives in small, pair-bonded groups that defend stable territories (5.47 ha) against neighboring conspecifics [65,68,70,71]. This territorial rigidity limits their ability to track shifting resource distributions since the area available is small and moving beyond its boundaries could result in competition with other *E. mongoz* groups. Similar patterns have been reported for other pair-living *Eulemur* species, such as *E. rubriventer*, which also maintain stable territories across seasons in eastern Madagascar [151]. Compared to sympatric *E. rufifrons*, *E. rubriventer* pairs occupy actively defended territories that vary little in size across seasonal periods [138,139,151]. If *E. mongoz* pairs face comparable constraints, persistence may be promoted by occupying sections of forest habitats where preferred resources are available across seasons [70,71,139,150]. This interpretation is consistent with the spatial pattern predicted in ANP and MCF: A higher probability of occurrence in lowland forest patches near water basins, where small, resource-rich territories are more likely to support stable pair-living groups – particularly during the dry season when vegetation moisture and surface water are most limited [70,121,125,150].

### 4.2. Patterns of niche overlap and divergence

Our analysis of niche equivalence between *E. fulvus* and *E. mongoz* reveals subtle, though statistically non-significant, differences in the breadth of environmental conditions associated with their niche requirements. Across both ANP and MCF, the occurrence of both species is primarily associated with lowland forest habitats characterized by high vegetation quality and proximity to surface water [55,70,73,140,152]. This shared preference for moist, green vegetation in continuous forest patches suggests a high degree of overlap in their Grinnellian niche dimensions [10,12]. This biogeographic association is supported by their predicted distribution patterns, which shows that most of the predicted geographic range of *E. mongoz* is also likely to contain *E. fulvus* groups. However, despite this overlap, the species differ in the specific topographic contexts where they are most frequently found. Consistent with the predicted geographic distributions, *E. mongoz* is more tightly associated with low-lying areas near water basins, while *E. fulvus* occupies a broader range of topographic positions. These differences likely reflect variation in spatial requirements and social organization: *E. fulvus* forms large, mobile groups with flexible home ranges [55,66,69], whereas *E. mongoz* defends small, stable territories [70,73,151]. As a result, although both species co-occur in similar vegetation zones, other dimensions of their niche ecology associated with their social organization, habitat use, and activity pattern may reduce direct competition and facilitate coexistence [12,29,65,67,153].

The lack of significant niche divergence between these two sympatric congeners suggests a high degree of niche conservatism – at least when assessed using broad-scale environmental variables [10,12,43,97]. This pattern is consistent with previous research on *Eulemur* species, which often exhibit substantial overlap in their ecological niches at regional scales [12,76,96,97,154,155]. *Eulemur* species tend to have a high proportion of niche overlap [53,67,76,96,97,155]. For example, among sympatric pairs of *Eulemur* species in Madagascar, all but one pair (*E. flavifrons* and *E. macaco*) exhibit no meaningful divergence in their niche requirements that could not be attributed to differences in their respective proportion of occupied habitats [96]. Instead, it is likely that for *Eulemur* species, differentiation of niche requirements between congeners may be based on more subtle distinctions in the functional relationship between each species and their environment – particularly for sympatric pairs. These results follow previous research on the evolution of the genus, suggesting that environmental filtering has played a dominant role in shaping the broad-scale distribution patterns of *Eulemur* species across Madagascar [53,96,97]; with both species persisting in forest areas that meet their shared environmental tolerances, at least in the short term. The high degree of similarity in their habitat tolerance, however, also suggests that competition might shape other aspects of each species’ niche not considered here – for example, their activity patterns and social organization [12,29,65,67,68].

Understanding the ecological mechanisms that allow *E. fulvus* and *E. mongoz* to coexist within lowland forest habitats in northwestern Madagascar likely requires a more nuanced, Eltonian perspective on niche differentiation – one that incorporates fine-scale behavioral and ecological interactions [10,12,30,155,156]. Indeed, previous research has shown that sympatric *Eulemur* often partition resources through differences in microhabitat use, foraging strategies, or activity patterns [67,155]. For example, *E. coronatus* and *E. sanfordi* in the north of Madagascar avoid direct competition by foraging at different forest strata and shifting their behavior during periods of resource scarcity [67,133]. Similar mechanisms may be at play between *E. fulvus* and *E. mongoz*, particularly in zones of co-occurrence where resource overlap is high. Temporal partitioning – such as shifts in activity patterns during the dry season – may be such a mechanism, reducing direct competition when food and canopy cover are limited [65,67,73]. Nevertheless, the high degree of niche overlap raises the possibility that *E. fulvus*, with its larger group sizes and broader spatial use, could competitively exclude *E. mongoz* from lower-quality habitats located farther from water basins. This dynamic may help explain the more restricted and fragmented distribution of *E. mongoz* across both landscapes. It also underscores the importance of future research on interspecific interactions and fine-scale resource partitioning, especially in light of ongoing habitat loss and climate change, which are likely to intensify competition and further reduce the availability of suitable habitats [157,158].

### 4.3. Conservation implications and future research

The variation in predicted occurrence patterns of *E. fulvus* and *E. mongoz* across ANP and MCF underscores that both species are forest specialists vulnerable to habitat loss and fragmentation. Although our analysis did not detect significant differences in their overall niche breadth, species-specific differences in habitat use suggest varying sensitivities to environmental conditions – particularly vegetation quality, moisture content, and topographic position. These differences are reflected in their realized distributions patterns across ANP and MCF, where *E. mongoz* occupies a smaller, more fragmented range than *E. fulvus*, a pattern that aligns with their respective conservation statuses [1,58,59]. We propose that differences in the realized niche space of each species – shaped by the combined envelope of their environmental preferences and other species-specific ecological traits like their social organization and ranging patterns – help explain the heightened extinction risk faced by *E. mongoz*.

However, here we should note that a key limitation of our analysis is that is that it only allows us to characterize habitat-associated components of the Grinnellian niche of *E. fulvus* and *E. mongoz* within ANP and MCF using largely static vegetation and topographic predictors. The absence of explicit bioclimatic variables (e.g., temperature and precipitation) prevents us from evaluating physiological tolerances and important climatic conditions that may structure patterns of occurrence and niche overlap at broader scales [76,153,157]. Because the degree by which the niche of any two species overlaps or divergences can differ markedly due to the type of factors that are considered [10,120,156], specially when evaluations do not incorporate relevant dimensions of an animal’s like climate, our landscape-only analysis can potentially under- or overestimate the fundamental degree to which the niche requirements of these two *Eulemur* species diverges from each other across their complete sympatric geographic range. This caveat is particularly relevant to our interpretation of conservation implications because our current predictions cannot disentangle whether differences in extinction risk reflect contrasting climate sensitivities, seasonal constraints in habitat use due to thermoregulation needs, or other factors not represented in our analysis [76,153,157].

Future work can address these limitations by expanding the spatial extent of our analysis beyond ANP and MCF. Leveraging increasingly available open-access occurrence datasets (e.g., research-grade records from iNaturalist and historic museum records), which are commonly used for biography and biodiversity research [76,159]. Modelling across a larger region would also allow the use of coarser, but climatically informative, datasets that can account for temperature and precipitation gradients, alongside temporally matched vegetation metrics (e.g., multi-year mean and interannual/seasonal variability such as the standard deviation of NDVI or related indices) that can account for long-term temporal distribution of occurrence records included in this expanded analysis [158,160,161]. Integrating long-term data on climate and vegetation dynamics with information on landscape structure and topography can improve comparability with previous lemur distribution studies and enable extrapolation of findings to other regions and climate-change scenarios [162–164]. Finally, an expanded spatiotemporal framework could allow us to test additional ecological mechanisms that account for the niche requirements of both species and explain differences in their vulnerability to extinction risk. If enough occurrence data is available, it is possible to contrast models fit to dry- and wet-season records, allowing us to test how thermal and hydration constraints influence the distribution patterns and realized niche requirements of both species [70,75,151,153].

Despite these limitations, our results provide meaningful insight on the ecology of these two species that helps us understand some of the variation in their current conservation risk. Showing that the broader and more continuous distribution of *E. fulvus* across lowland forests enhances its ecological viability by increasing habitat connectivity and access to key resources [1,24,27,38,165]. Relative to *E. mongoz*, the flexible niche requirements of *E. fulvus* allow it to exploit forest habitats of varying vegetation quality, adapting its group size, foraging strategies, and movement patterns to match local conditions and maximize their access to preferred resources [66,69,131,137,141]. Moreover, this behavioral and demographic flexibility has likely buffered *E. fulvus* against the effects of habitat degradation in ANP and MCF [64,69]. While further research is needed to determine the effectiveness of short- and long-range dispersal across these two fragmented landscapes [36,55,166], models of animal dispersal suggest that the large area and continuity of their distribution patterns increase the likelihood of successful dispersal and gene flow, reducing demographic isolation and extinction risk [24,25,38].

In contrast, the specific niche requirements of *E. mongoz*, combined with its strict behavioral and demographic traits, restrict its distribution to moist lowland sections of forest patches near water basins. This narrow tolerance of environmental conditions limits the availability of suitable habitat and increases how fragmentated the population is in these landscapes, making this species more vulnerable to environmental change [25,26,70,73]. Moreover, while both *Eulemur* species likely encounter similar physical barriers to dispersal, *E. mongoz* faces disproportionately higher energetic and demographic costs due to the combined effects of its fragmented distribution and socially constrained mating system [25–27,167]. Living in pair-bonded groups with small, defended territories, *E. mongoz* individuals must overcome not only the challenge of locating suitable microhabitats, but also the additional burden of finding conspecifics who are both reproductively available and genetically appropriate [24,25,168,169]. Crucially, these two requirements – habitat suitability and mate availability – do not necessarily coincide spatially, further compounding the difficulty of successful dispersal and reproduction compared to the more socially flexible *E. fulvus* [73,170–172].

More to this point, the social characteristics of primates (e.g., group size and social system) can amplify their intrinsic vulnerability to extinction. Species like *E. mongoz*, which form small, pair-bonded groups, are particularly susceptible because their reproductive potential is tightly linked to population density and a balanced sex ratio. In such systems, even modest declines in population size or disruptions in group composition can severely limit reproductive opportunities [171–174]. In contrast, *E. fulvus* lives in larger, multi-male multi-female groups with more flexible social structures, allowing for greater tolerance of demographic fluctuations. For *E. fulvus*, reproductive success depends primarily on the presence of both sexes, rather than on strict pair bonds, making it easier for dispersing individuals to integrate into new groups and reproduce [170,173,174]. These differences in social organization, when combined with species-specific niche requirements, shape how each species navigates fragmented landscapes and interacts with conspecifics. Together, they determine the level of effort required to meet fundamental ecological needs – that is, accessing suitable habitat and securing reproductive opportunities [25–27,174]. It is likely then, that the fragmented configuration of preferred habitats across ANP and MCF appear to affect the capacity of *E. mongoz* populations to meet these requirements more severely than it does for *E. fulvus* populations, increasing their relative likelihood of extinction and explaining why *E. fulvus* groups are more commonly present in these landscapes than *E. mongoz* groups.

### 4.4. Conclusion

In conclusion, our findings demonstrate that even subtle differences in the niche requirements of closely related, sympatric species can lead to substantial variation in their distribution patterns and conservation vulnerability. For sympatric *E. mongoz* and *E. fulvus*, differences in niche flexibility, habitat preference, and social structure translate into markedly different responses to habitat loss, fragmentation, and degradation [5,6]. These differences help explain the contrasting patterns of distribution, local abundance, and extinction risk observed across their shared ranges in northwestern Madagascar [55,58–60,62,65]. Our results suggest that environmental filtering plays an important role in shaping the broad-scale distributions of both species across lowland, dry-deciduous forests. At finer spatial scales, niche partitioning – driven by differences in foraging behavior, activity patterns, and social organization – enables *E. fulvus* to exploit a wider range of forest conditions than *E. mongoz* while potentially reducing direct competition in areas of habitat overlap [55,67,69,70,73]. However, these same traits that facilitate coexistence also constrain *E. mongoz*’*s* ability to adapt to environmental change. Its narrow niche and social system limit its capacity to disperse, reproduce, and persist in fragmented landscapes [5,6,174]. Together, these ecological and demographic constraints illustrate how the niche characteristics of any species shape their extinction risk – determining which species can persist as habitats continue to degrade.

The successful protection of *E. mongoz* will ultimately depend on how effectively conservation efforts safeguard the extent and ecological integrity of lowland forests that support its preferred moist and evergreen vegetation [59,64,73]. These habitats are critical for maintaining year-round resource availability but are also among the most vulnerable to anthropogenic pressures [62,64,175–177]. Their proximity to surface water makes them highly attractive for human settlement and agricultural expansion, intensifying the risk of habitat loss and fragmentation. In contrast, *E. fulvus* demonstrates greater ecological flexibility, allowing it to persist in a wider range of forest conditions. However, its continued presence across these landscapes still depends on the availability of sufficient forest area and canopy cover [55,69]. To ensure the long-term viability of *E. fulvus* populations, conservation strategies must prioritize forest restoration efforts that increase habitat area and connectivity – specially outside the boundaries of ANP and MCF, where forest cover is more fragmented and degraded [62,64].

## Acknowledgement

The authors would like to thank the Ministère de l’Environnement et du Developpement Durable, the Direction du Système des Aires Protégées, and Madagascar National Parks for permission to conduct research in ANP and MCF. We are grateful to Dr. Romule Rakotondravony, Dr. Ute Radespiel, Dr. Frederik Kiene, Malcolm S. Ramsay, the organizations Planet Madagascar, DBCAM, Operation Wallacea, and the people of the villages of Maevatanimbary, Andranohobaka, Ambarindahy, Mariarano, Ambolodihy, and Antanambao for their technical and logistical support. We would also like to thank the anonymous reviewers for their valuable comments and suggestions, which helped to improve the quality of this manuscript.

## Supporting information

S1 Fig Locations of communities, research stations, and survey sites in the vicinity of areas surveyed for E. fulvus and E. mongoz occurrences during the 2019 field season. The basemap is USGS/NASA Landsat 8 Level-2 surface reflectance imagery (LC08_L2SP_160071_2019 0601_20200828_02_T1). Protected-area boundary for ANP digitized by the authors from an official map by Madagascar National Parks and the Ministry of Environment that was provided during fieldwork (georeferenced using field control points); MCF boundary delineated by the authors to encompass surveyed forest patches. The RN4 highway and the locations of survey sites were digitized by the first author from handheld Garmin GPS waypoint and track data collected during fieldwork.
(PDF)

S2 Fig Model evaluation: omission, predicted AUC, and ROC curves for E. mongoz and E. fulvus. Panels A and C include the average Omission and predicted area under the curve (AUC) for E. mongoz and E. fulvus respectively. Panels C and D include receiver-operating characteristic curves (ROC) for E. mongoz and E. fulvus, respectively.
(PDF)

S3 Fig Boxplots of niche equivalence and divergence tests for E. mongoz and E. fulvus (PC1, PC2). Panels A and B include boxplots of each species niche equivalence comparison for our niche overlap (NOT) and niche divergence (NDT) tests. Each boxplot presents one dimension (PC1 and PC2) of the total and overlapping E-space occupied by each species.
(PDF)

S1 Table Description of candidate environmental covariates. Description of candidate environmental covariates included in our analysis of E. fulvus and E. mongoz distribution patterns and niche equivalence.
(PDF)

S2 Table Autocorrelation matrix for environmental variables used in MaxEnt models. Autocorrelation matrix for the 10 background environmental variables used in our Eulemur fulvus and E. mongoz MaxEnt models. Combinations of the first eight variables were performed using Spearman’s test for autocorrelation. For the two categorical variables*, we first used point-biserial correlation to compare the first eight continuous variables against the binary Forest Cover (FRST) variable. Then, we used the correlation ratio (η^2^) to compare the eight continuous variables to the multiclass Habitat Class variable (HBT). Finally, we used Cramér’s V to measure the strength of association between the Forest Cover and Habitat Class variables. Whenever we observed a high correlation between pairs of candidate variables, their correlation estimate was reported in bold.
(PDF)

S3 Table Top five MaxEnt model implementations from spatial jackknife. Top five E. fulvus and E. mongoz SDM implementations evaluated during the spatial jackknife training and validation protocol. SDM implementations included alternative model parametrizations, testing a range of regularization multipliers and feature classes. Model performance was evaluated by first comparing test of omission rates (OR) and area under the curve (AUC) estimates for each candidate run, selecting runs with the lowest OR and highest AUC. If the top candidate models exhibited comparable OR and AUC estimates, we selected the model with the simplest feature class. From simplest to most complex, possible feature classes include linear (L); linear and quadratic (LQ); hinge (H); linear, quadratic, and hinge (LQH); linear, quadratic, hinge, product, and threshold (LQHPT).
(PDF)

S4 Table Partial ROC evaluation summary for final E. mongoz MaxEnt model. Partial ROC evaluation summary for final E. mongoz MaxEnt model within the accessible area M under five different random-seed allocations to the 80−20 random training data split. Partial ROC ratio and 95% bootstrap CI focus on the high-sensitivity (low-omission) region defined by E and summarizes performance as an AUC ratio. Also present is a summary of the parameters for the evaluation and an estimate of the full AUC value for each species’ model.
(PDF)

S5 Table Key to interpreting’Humboldt’ results. **significant test, NS = non-significant test, *a significant divergence test could equally be reflective of differences in favorable biotic factors between test regions with equal fundamental niches. The biotic factors can include the identity and abundance of facilitators (e.g., pollinators, seed dispersers), predators, parasites, pathogens, and competitors that constrain or facilitate a species distribution (Gaston, 2003). Table was reproduced from Brown and Carnaval, 2019.
(PDF)
