# Supplementary material for: Differentiation of ecological niche patterns between sympatric lemurs in northwestern Madagascar: Implications for their conservation
Source: PLoS One. 2026 Mar 19;21(3):e0345256. doi: 10.1371/journal.pone.0345256 (PMC13001921; doi:10.1371/journal.pone.0345256)
Supplement: S1 Table — Description of candidate environmental covariates included in our analysis of E. fulvus and E. mongoz distribution patterns and niche equivalence. (PDF) [file pone.0345256.s004.pdf]

Full Title: Differentiation of ecological niche patterns between sympatric lemurs in northwestern Madagascar: Implications for their conservation

Short Title: Differences in ecological niche patterns of sympatric lemurs

Authors: Fernando Mercado Malabet[1#]*, Finaritra T. Randimbiarison[2¶], Jean Claude Razafimampiandra[2¶], Bertrand Andriatsitohaina[3,5&], Coral Chell[1], Mamy Razafitsalama[5&], Travis S. Steffens[4,5], and Shawn M. Lehman[1]

[1] Department of Anthropology, University of Toronto, Toronto, Ontario, Canada.

[2] Mention Zoologie et Biodiversité Animale, Université d'Antananarivo, Antananarivo 101, Madagascar.

[3] Faculté des Sciences, de Technologies et de l'Environnement, Université de Mahajanga, Mahajanga, Madagascar.

[4] Department of Sociology and Anthropology, University of Guelph, Guelph, Ontario, Canada.

[5] Planet Madagascar, Guelph, Ontario, Canada.

[#] Current Address: Department of Ecosystem Science and Management, University of Northern British Columbia, Prince George, British Columbia, Canada.

* Corresponding Author: Fernando Mercado Malabet

Email: fernando.mercadomalabet@mail.utoronto.ca

¶ These authors contributed equally to this work.

& These authors also contributed equally to this work.

Abstract: Understanding how species respond to habitat loss and fragmentation is a critical requirement for effective conservation action, particularly in biodiversity hotspots like Madagascar. Species with specialized, narrower ecological niche requirements are hypothesized to be more vulnerable to extinction than generalists, yet empirical tests of this prediction among closely related taxa remain limited. Here, we compare the ecological niche patterns and predicted distributions of two sympatric lemurs in northwestern Madagascar – the Vulnerable Common Brown Lemur (*Eulemur fulvus*) and the Critically Endangered Mongoose Lemur (*Eulemur mongoz*) – to assess how niche flexibility relates to extinction risk. Using presence-only data collected between 2015 and 2020 and ten environmental covariates, we developed species distribution models and ran niche equivalence analysis. The models indicate that *E. fulvus* occupies a broader and more continuous predicted distribution range (48,591 ha) than *E. mongoz* (17,757 ha). In comparison, *E. mongoz* is predicted to occur primarily in moist lowland forests near water basins, showing a stronger spatial association with these habitat conditions that *E. fulvus*. Despite these marked differences in their predicted geographic distributions, niche equivalence analysis showed substantial overlap in the environmental conditions occupied by the two species within the study area. Together, these results suggest that *E. mongoz's* restricted distribution is not explained solely by the measured environmental predictors, highlighting the need for future work that integrates additional environmental variables and evaluates potential behavioural or demographic constraints not captured here. These findings highlight how subtle differences in niche requirements can shape a species' habitat use and vulnerability to environmental change. From a management perspective, our findings support prioritizing the protection of moist lowland forests near water basins for *E. mongoz* while maintaining or enhancing habitat connectivity for *E. fulvus* in fragmented landscapes.

Supporting Information:

S1 Table. Description of candidate environmental covariates. *Description of candidate environmental covariates included in our analysis of E. fulvus and E. mongoz distribution patterns and niche equivalence.*

Name	Description	Reference
Landcover Class	Indicates the distribution of landcover classes across ANP and MCF. This variable was calculated using the Maximum Likelihood Supervised Classification tool from the Spatial Analyst extension in ArcGIS desktop v10.8.1 to classify Landsat 8 Level 2 multispectral imagery from the summer of 2019. Landcover classes included surface water bodies, sandy banks, grassland, brush thicket, seasonal dry forest, evergreen forest, classified with values ranging from 1 to 6 respectively.	ESRI, 2020; USGS, 2019; Vermote et al. 2016
Forest Cover	A reclassified version of the *Landcover Class* variable, indicating the distribution of forest habitats across ANP and MCF. This variable was calculated using the reclassify tool from the Spatial Analyst extension in ArcGIS desktop v10.8.1. Landcover classes with values between 1 and 4 were reclassified as Matrix class, with a value of 0. Landcover classes with values between 5 and 6 were classified as Forest class, with a value of 1.	ESRI, 2020
Forest Cover Area (m^2)	Indicates the area (m^2) of forest habitat as determined from our *Forest Cover* variable. This variable was calculated using the patch area (lsm_p_area) function from the *landscapemetrics v1.5.4* in R v4.1.3	Hasselbarth et al., 2019
Forest Cover Change (2000 to 2021)	Indicates changes in forest cover between 2000 to 2021, with values ranging between 0 and 21. Pixels with a value of 2021 indicate that no changes in forest cover had taken place until 2021. Lower values indicate some change in forest cover during the respective year (2000 to 2020). Useful for mapping temporal patterns of habitat degradation.	Hansen et al., 2013

Distance to water basin (m)	The Euclidean distance (m) from open bodies of water. This variable was calculated by reclassifying the *MNDWI* variable to water and non-water features, based on the distribution of pixel values above and below 0. Then, using the reclassified variable, we calculated the distance of all pixels in the non-water category to the closest water feature by running the Euclidean Distance tool from the Spatial Analyst extension in ArcGIS desktop v10.8.1.	ESRI, 2020; Xu2006
Elevation (m)	Elevation (m) from sea level data was obtained from NASA's Shuttle Radar Topography Mission (SRTM) void-filled Digital Elevation Model (DEM). This DEM offers a worldwide coverage of elevation data at a resolution of 1 arc-second (30 m), void-filled with the ASTER GDEM and USGS GMTED2010 datasets.	Farr et al., 2007; NASA SRTM, 2013
Slope (%)	Calculated from the SRTM DEM, this variable identifies the rise in elevation from one DEM cell to the next as a percent value, ranging from 0 to infinity. A value of 0 indicates a flat surface and greater percentage rise values indicate more vertical inclinations. This variable was calculated using the Slope tool from the Spatial Analyst extension in ArcGIS desktop v10.8.1.	ESRI, 2020
Normalized Difference Vegetation Index (NDVI)	Identifies the amount of aboveground vegetation greenness based on its primary productivity. Useful for understanding vegetation density and assessing spatial variation in vegetation quality. Values closer to 1 indicate higher vegetation quality. This variable was calculated from Landsat 8 Level 2 multispectral imagery, using the Raster Calculator tool from the Spatial Analyst extension in ArcGIS desktop v10.8.1.	ESRI, 2020; Landsat Missions, n.d.; USGS, 2019
Normalized Difference Moisture Index (NDMI)	Identifies the amount of water content in vegetation. Useful for understanding patterns of vegetation moisture. Values closer to 1 indicate higher vegetation moisture. This variable was calculated from Landsat 8 Level 2 multispectral imagery, using the Raster Calculator tool from	ESRI, 2020; Landsat Missions, n.d.; USGS, 2019

	the Spatial Analyst extension in ArcGIS desktop v10.8.1.	
Modified Normalized Difference Water Index (MNDWI)	Identifies open bodies of standing and flowing water while diminishing the confounding albedo effect of built-up area features. Useful for mapping the distribution of waterbodies in a landscape. Values above 0 indicate open water content, with values close to 1 indicating water bodies with greater depth and clarity. This variable was calculated from Landsat 8 Level 2 multispectral imagery, using the Raster Calculator tool from the Spatial Analyst extension in ArcGIS desktop v10.8.1.	ESRI, 2020; USGS, 2019; Xu, 2006
