# Supplementary material for: Differentiation of ecological niche patterns between sympatric lemurs in northwestern Madagascar: Implications for their conservation
Source: PLoS One. 2026 Mar 19;21(3):e0345256. doi: 10.1371/journal.pone.0345256 (PMC13001921; doi:10.1371/journal.pone.0345256)
Supplement: S2 Table — Autocorrelation matrix for the 10 background environmental variables used in our Eulemur fulvus and E. mongoz MaxEnt models. Combinations of the first eight variables were performed using Spearman’s test for autocorrelation. For the two categorical variables*, we first used point-biserial correlation to compare the first eight continuous variables against the binary Forest Cover (FRST) variable. Then, we used the correlation ratio (η2) to compare the eight continuous variables to the multiclass Habitat Class variable (HBT). Finally, we used Cramér’s V to measure the strength of association between the Forest Cover and Habitat Class variables. Whenever we observed a high correlation between pairs of candidate variables, their correlation estimate was reported in bold. (PDF) [file pone.0345256.s005.pdf]

Full Title: Differentiation of ecological niche patterns between sympatric lemurs in northwestern Madagascar: Implications for their conservation

Short Title: Differences in ecological niche patterns of sympatric lemurs

Authors: Fernando Mercado Malabet[1#]*, Finaritra T. Randimbiarison[2¶], Jean Claude Razafimampiandra[2¶], Bertrand Andriatsitohaina[3,5&], Coral Chell[1], Mamy Razafitsalama[5&], Travis S. Steffens[4,5], and Shawn M. Lehman[1]

[1] Department of Anthropology, University of Toronto, Toronto, Ontario, Canada.

[2] Mention Zoologie et Biodiversité Animale, Université d'Antananarivo, Antananarivo 101, Madagascar.

[3] Faculté des Sciences, de Technologies et de l'Environnement, Université de Mahajanga, Mahajanga, Madagascar.

[4] Department of Sociology and Anthropology, University of Guelph, Guelph, Ontario, Canada.

[5] Planet Madagascar, Guelph, Ontario, Canada.

[#] Current Address: Department of Ecosystem Science and Management, University of Northern British Columbia, Prince George, British Columbia, Canada.

* Corresponding Author: Fernando Mercado Malabet

Email: fernando.mercadomalabet@mail.utoronto.ca

¶ These authors contributed equally to this work.

& These authors also contributed equally to this work.

Abstract: Understanding how species respond to habitat loss and fragmentation is a critical requirement for effective conservation action, particularly in biodiversity hotspots like Madagascar. Species with specialized, narrower ecological niche requirements are hypothesized to be more vulnerable to extinction than generalists, yet empirical tests of this prediction among closely related taxa remain limited. Here, we compare the ecological niche patterns and predicted distributions of two sympatric lemurs in northwestern Madagascar – the Vulnerable Common Brown Lemur (*Eulemur fulvus*) and the Critically Endangered Mongoose Lemur (*Eulemur mongoz*) – to assess how niche flexibility relates to extinction risk. Using presence-only data collected between 2015 and 2020 and ten environmental covariates, we developed species distribution models and ran niche equivalence analysis. The models indicate that *E. fulvus* occupies a broader and more continuous predicted distribution range (48,591 ha) than *E. mongoz* (17,757 ha). In comparison, *E. mongoz* is predicted to occur primarily in moist lowland forests near water basins, showing a stronger spatial association with these habitat conditions that *E. fulvus*. Despite these marked differences in their predicted geographic distributions, niche equivalence analysis showed substantial overlap in the environmental conditions occupied by the two species within the study area. Together, these results suggest that *E. mongoz's* restricted distribution is not explained solely by the measured environmental predictors, highlighting the need for future work that integrates additional environmental variables and evaluates potential behavioural or demographic constraints not captured here. These findings highlight how subtle differences in niche requirements can shape a species' habitat use and vulnerability to environmental change. From a management perspective, our findings support prioritizing the protection of moist lowland forests near water basins for *E. mongoz* while maintaining or enhancing habitat connectivity for *E. fulvus* in fragmented landscapes.

Supporting Information:

S2 Table. Autocorrelation matrix for environmental variables used in MaxEnt models. *Autocorrelation matrix for the 10 background environmental variables used in our Eulemur fulvus and E. mongoz MaxEnt models. Combinations of the first eight variables were performed using Spearman's test for autocorrelation. For the two categorical variables*, we first used point-biserial correlation to compare the first eight continuous variables against the binary Forest Cover (FRST) variable. Then, we used the correlation ratio (η^2) to compare the eight continuous variables to the multiclass Habitat Class variable (HBT). Finally, we used Cramér's V to measure the strength of association between the Forest Cover and Habitat Class variables. Whenever we observed a high correlation between pairs of candidate variables, their correlation estimate was reported in bold.*

	**ELEV**	**SLOPE**	**FAREA**	**FLOSS**	**NDVI**	**NDMI**	**MNDWI**	**DTWB**	**FRST***	**HBT****
**ELEV**	**1**	-0.01	0.24	0.46	0.15	0.13	0.16	0.40	0.30	0.10
**SLOPE**	-0.01	**1**	-0.01	-0.03	-0.05	-0.05	-0.03	0.02	-0.02	0.00
**FAREA**	0.24	-0.01	**1**	**0.83**	**0.76**	**0.79**	**0.75**	-0.03	**0.96**	**0.54**
**FLOSS**	0.46	-0.03	**0.83**	**1**	**0.72**	**0.70**	0.64	-0.00	**0.75**	**0.88**
**NDVI**	0.15	-0.05	**0.76**	**0.72**	**1**	**0.95**	0.69	-0.08	**0.83**	**0.75**
**NDMI**	0.13	-0.05	**0.79**	**0.70**	**0.95**	**1**	**0.85**	-0.09	**0.83**	**0.74**
**MNDWI**	0.16	-0.03	**0.75**	0.64	0.69	**0.85**	**1**	-0.06	**0.67**	**0.51**
**DTWB**	0.40	0.02	-0.03	-0.00	-0.08	-0.09	-0.06	**1**	-0.03	0.01
**FRST***	0.30	-0.02	**0.96**	**0.75**	**0.83**	**0.83**	**0.67**	-0.03	**1**	**0.98**
**HBT****	0.10	0.00	**0.54**	**0.88**	**0.75**	**0.74**	**0.51**	0.01	**0.98**	**1**

NOTE: Thresholds of high correlation among our candidate variables should be interpreted as follows.

- Spearman's ρ for continuous-continuous ≥ 0.7.
- Point-Biserial for continuous-binary $|r| \geq 0.5$.
- Correlation Ratio for continuous-multiclass (η^2) ≥ 0.3 [moderate] or ≥ 0.5 [strong].
- Cramér's V for categorical-categorical ≥ 0.3 [moderate] or ≥ 0.5 [strong].
