# Supplementary material for: Differentiation of ecological niche patterns between sympatric lemurs in northwestern Madagascar: Implications for their conservation
Source: PLoS One. 2026 Mar 19;21(3):e0345256. doi: 10.1371/journal.pone.0345256 (PMC13001921; doi:10.1371/journal.pone.0345256)
Supplement: S3 Table — Top five E. fulvus and E. mongoz SDM implementations evaluated during the spatial jackknife training and validation protocol. SDM implementations included alternative model parametrizations, testing a range of regularization multipliers and feature classes. Model performance was evaluated by first comparing test of omission rates (OR) and area under the curve (AUC) estimates for each candidate run, selecting runs with the lowest OR and highest AUC. If the top candidate models exhibited comparable OR and AUC estimates, we selected the model with the simplest feature class. From simplest to most complex, possible feature classes include linear (L); linear and quadratic (LQ); hinge (H); linear, quadratic, and hinge (LQH); linear, quadratic, hinge, product, and threshold (LQHPT). (PDF) [file pone.0345256.s006.pdf]

Full Title: Differentiation of ecological niche patterns between sympatric lemurs in northwestern Madagascar: Implications for their conservation

Short Title: Differences in ecological niche patterns of sympatric lemurs

Authors: Fernando Mercado Malabet[1#]*, Finaritra T. Randimbiarison[2¶], Jean Claude Razafimampiandra[2¶], Bertrand Andriatsitohaina[3,5&], Coral Chell[1], Mamy Razafitsalama[5&], Travis S. Steffens[4,5], and Shawn M. Lehman[1]

[1] Department of Anthropology, University of Toronto, Toronto, Ontario, Canada.

[2] Mention Zoologie et Biodiversité Animale, Université d'Antananarivo, Antananarivo 101, Madagascar.

[3] Faculté des Sciences, de Technologies et de l'Environnement, Université de Mahajanga, Mahajanga, Madagascar.

[4] Department of Sociology and Anthropology, University of Guelph, Guelph, Ontario, Canada.

[5] Planet Madagascar, Guelph, Ontario, Canada.

[#] Current Address: Department of Ecosystem Science and Management, University of Northern British Columbia, Prince George, British Columbia, Canada.

* Corresponding Author: Fernando Mercado Malabet

Email: fernando.mercadomalabet@mail.utoronto.ca

¶ These authors contributed equally to this work.

& These authors also contributed equally to this work.

Abstract: Understanding how species respond to habitat loss and fragmentation is a critical requirement for effective conservation action, particularly in biodiversity hotspots like Madagascar. Species with specialized, narrower ecological niche requirements are hypothesized to be more vulnerable to extinction than generalists, yet empirical tests of this prediction among closely related taxa remain limited. Here, we compare the ecological niche patterns and predicted distributions of two sympatric lemurs in northwestern Madagascar – the Vulnerable Common Brown Lemur (*Eulemur fulvus*) and the Critically Endangered Mongoose Lemur (*Eulemur mongoz*) – to assess how niche flexibility relates to extinction risk. Using presence-only data collected between 2015 and 2020 and ten environmental covariates, we developed species distribution models and ran niche equivalence analysis. The models indicate that *E. fulvus* occupies a broader and more continuous predicted distribution range (48,591 ha) than *E. mongoz* (17,757 ha). In comparison, *E. mongoz* is predicted to occur primarily in moist lowland forests near water basins, showing a stronger spatial association with these habitat conditions that *E. fulvus*. Despite these marked differences in their predicted geographic distributions, niche equivalence analysis showed substantial overlap in the environmental conditions occupied by the two species within the study area. Together, these results suggest that *E. mongoz's* restricted distribution is not explained solely by the measured environmental predictors, highlighting the need for future work that integrates additional environmental variables and evaluates potential behavioural or demographic constraints not captured here. These findings highlight how subtle differences in niche requirements can shape a species' habitat use and vulnerability to environmental change. From a management perspective, our findings support prioritizing the protection of moist lowland forests near water basins for *E. mongoz* while maintaining or enhancing habitat connectivity for *E. fulvus* in fragmented landscapes.

Supporting Information:

S3 Table. Top five MaxEnt model implementations from spatial jackknife. *Top five E. fulvus and E. mongoz SDM implementations evaluated during the spatial jackknife training and validation protocol. SDM implementations included alternative model parametrizations, testing a range of regularization multipliers and feature classes. Model performance was evaluated by first comparing test of omission rates (OR) and area under the curve (AUC) estimates for each candidate run, selecting runs with the lowest OR and highest AUC. If the top candidate models exhibited comparable OR and AUC estimates, we selected the model with the simplest feature class. From simplest to most complex, possible feature classes include linear (L); linear and quadratic (LQ); hinge (H); linear, quadratic, and hinge (LQH); linear, quadratic, hinge, product, and threshold (LQHPT).*

Reg. Multiplier	Feature Class	Weighted OR	AUC	OR & AUC
		Eulemur fulvus		
6.0	LQ	0.8936	0.7403	1.6339
4.0	LQ	0.8917	0.7425	1.6342
6.0	LQH	0.8913	0.7525	1.6437
5.0	L	0.8912	0.7345	1.6258
1.5	LQ	0.8909	0.7566	1.6375
		Eulemur mongoz		
6.0	H	0.8782	0.8830	1.7612
4.0	H	0.8757	0.8864	1.7621
6.0	L	0.8754	0.8911	1.7665
5.0	L	0.8741	0.8913	1.7654
4.0	L	0.8730	0.8943	1.7673
