# Supplementary material for: Differentiation of ecological niche patterns between sympatric lemurs in northwestern Madagascar: Implications for their conservation
Source: PLoS One. 2026 Mar 19;21(3):e0345256. doi: 10.1371/journal.pone.0345256 (PMC13001921; doi:10.1371/journal.pone.0345256)
Supplement: S5 Table — **significant test, NS = non-significant test, *a significant divergence test could equally be reflective of differences in favorable biotic factors between test regions with equal fundamental niches. The biotic factors can include the identity and abundance of facilitators (e.g., pollinators, seed dispersers), predators, parasites, pathogens, and competitors that constrain or facilitate a species distribution (Gaston, 2003). Table was reproduced from Brown and Carnaval, 2019. (PDF) [file pone.0345256.s008.pdf]

Full Title: Differentiation of ecological niche patterns between sympatric lemurs in northwestern Madagascar: Implications for their conservation

Short Title: Differences in ecological niche patterns of sympatric lemurs

Authors: Fernando Mercado Malabet[1#*], Finaritra T. Randimbiarison[2¶], Jean Claude Razafimampiandra[2¶], Bertrand Andriatsitohaina[3,5&], Coral Chell[1], Mamy Razafitsalama[5&], Travis S. Steffens[4,5], and Shawn M. Lehman[1]

[1] Department of Anthropology, University of Toronto, Toronto, Ontario, Canada.

[2] Mention Zoologie et Biodiversité Animale, Université d'Antananarivo, Antananarivo 101, Madagascar.

[3] Faculté des Sciences, de Technologies et de l'Environnement, Université de Mahajanga, Mahajanga, Madagascar.

[4] Department of Sociology and Anthropology, University of Guelph, Guelph, Ontario, Canada.

[5] Planet Madagascar, Guelph, Ontario, Canada.

[#] Current Address: Department of Ecosystem Science and Management, University of Northern British Columbia, Prince George, British Columbia, Canada.

* Corresponding Author: Fernando Mercado Malabet

Email: fernando.mercadomalabet@mail.utoronto.ca

¶ These authors contributed equally to this work.

& These authors also contributed equally to this work.

Abstract: Understanding how species respond to habitat loss and fragmentation is a critical requirement for effective conservation action, particularly in biodiversity hotspots like Madagascar. Species with specialized, narrower ecological niche requirements are hypothesized to be more vulnerable to extinction than generalists, yet empirical tests of this prediction among closely related taxa remain limited. Here, we compare the ecological niche patterns and predicted distributions of two sympatric lemurs in northwestern Madagascar – the Vulnerable Common Brown Lemur (*Eulemur fulvus*) and the Critically Endangered Mongoose Lemur (*Eulemur mongoz*) – to assess how niche flexibility relates to extinction risk. Using presence-only data collected between 2015 and 2020 and ten environmental covariates, we developed species distribution models and ran niche equivalence analysis. The models indicate that *E. fulvus* occupies a broader and more continuous predicted distribution range (48,591 ha) than *E. mongoz* (17,757 ha). In comparison, *E. mongoz* is predicted to occur primarily in moist lowland forests near water basins, showing a stronger spatial association with these habitat conditions that *E. fulvus*. Despite these marked differences in their predicted geographic distributions, niche equivalence analysis showed substantial overlap in the environmental conditions occupied by the two species within the study area. Together, these results suggest that *E. mongoz's* restricted distribution is not explained solely by the measured environmental predictors, highlighting the need for future work that integrates additional environmental variables and evaluates potential behavioural or demographic constraints not captured here. These findings highlight how subtle differences in niche requirements can shape a species' habitat use and vulnerability to environmental change. From a management perspective, our findings support prioritizing the protection of moist lowland forests near water basins for *E. mongoz* while maintaining or enhancing habitat connectivity for *E. fulvus* in fragmented landscapes.

Supporting Information:

S5 Table. Key to interpreting 'Humboldt' results. ***significant test, NS= non-significant test, *a significant divergence test could equally be reflective of differences in favorable biotic factors between test regions with equal fundamental niches. The biotic factors can include the identity and abundance of facilitators (e.g., pollinators, seed dispersers), predators, parasites, pathogens, and competitors that constrain or facilitate a species distribution (Gaston, 2003). Table was reproduced from Brown and Carnaval, 2019.*

Niche Overlap Test		Niche Divergence Test		Interpretation
**Equivalence Statistic**	**Background Statistic**	**Equivalence Statistic**	**Background Statistic**	
**	** or NS	**	** or NS	Strong evidence that the species' niches have diverged*
**	** or NS	Not Possible – No Overlap	-	Strong evidence that the species' niches have diverged*
NS	**	**	**	The species' niches have diverged; however, there are no significant differences when looking at total distribution. Typically, this result suggests the relationship in the NDT is marginally significant (0.05 < P < 0.1). This can occur if data were not properly rarefied, or environmental correlations need to be removed from the niches of species.
**	**	NS	**	This supports the hypothesis that their current niches are not equivalent. However, these results do not support that the hypothesis is the result of divergent evolution; rather, differences are simply a result of different access to environments.
NS	NS	**	** or NS	Evidence that the species' niches have diverged*. In this case, the NOT is not significant in both the Equivalence statistic and Background statistic and is

				inconclusive. The NOT Background statistic suggests that the E-space available is similar to one or both distributions. Because of this, there is limited power to detect a difference even if one exists.
**	** or NS	NS	NS	This supports the hypothesis that their current niches are not equivalent. However, these results do not support that the hypothesis is the result of divergent evolution; rather, differences are simply a result of different access to environments. The NDT Background statistic suggests that the E-space of one species is similar to one or both species occupied distributions. Because of this, there is limited power to detect a difference even if one exists.
NS	NS	NS	NS	These results are inconclusive but suggests the niches are equivalent. However, this may be due to limited E-space available in both distributions.
NS	**	NS	**	Strong evidence that the niches are equivalent
